# Dichotomous Role of Tumor Necrosis Factor in Pulmonary Barrier Function and Alveolar Fluid Clearance

**DOI:** 10.3389/fphys.2021.793251

**Published:** 2022-02-21

**Authors:** Rudolf Lucas, Yalda Hadizamani, Perenlei Enkhbaatar, Gabor Csanyi, Robert W. Caldwell, Harald Hundsberger, Supriya Sridhar, Alice Ann Lever, Martina Hudel, Dipankar Ash, Masuko Ushio-Fukai, Tohru Fukai, Trinad Chakraborty, Alexander Verin, Douglas C. Eaton, Maritza Romero, Jürg Hamacher

**Affiliations:** ^1^Vascular Biology Center, Augusta University, Augusta, GA, United States; ^2^Department of Pharmacology and Toxicology, Augusta University, Augusta, GA, United States; ^3^Department of Medicine, Medical College of Georgia, Augusta University, Augusta, GA, United States; ^4^Lungen-und Atmungsstiftung Bern, Bern, Switzerland; ^5^Pneumology, Clinic for General Internal Medicine, Lindenhofspital Bern, Bern, Switzerland; ^6^Department of Anesthesiology, University of Texas Medical Branch, Galveston, TX, United States; ^7^Department of Medical Biotechnology, University of Applied Sciences, Krems, Austria; ^8^Department of Dermatology, University Hospital of the Paracelsus Medical University, Salzburg, Austria; ^9^Institute for Medical Microbiology, Justus-Liebig University, Giessen, Germany; ^10^Charlie Norwood Veterans Affairs Medical Center, Augusta, GA, United States; ^11^Department of Medicine, School of Medicine, Emory University, Atlanta, GA, United States; ^12^Department of Anesthesiology and Perioperative Medicine, Medical College of Georgia, Augusta University, Augusta, GA, United States; ^13^Medical Clinic V-Pneumology, Allergology, Intensive Care Medicine, and Environmental Medicine, Faculty of Medicine, University Medical Centre of the Saarland, Saarland University, Homburg, Germany; ^14^Institute for Clinical & Experimental Surgery, Faculty of Medicine, Saarland University, Homburg, Germany

**Keywords:** TNF receptor, TNF lectin-like domain, acute respiratory distress syndrome, COVID-19, epithelial sodium channel

## Abstract

Alveolar-capillary leak is a hallmark of the acute respiratory distress syndrome (ARDS), a potentially lethal complication of severe sepsis, trauma and pneumonia, including COVID-19. Apart from barrier dysfunction, ARDS is characterized by hyper-inflammation and impaired alveolar fluid clearance (AFC), which foster the development of pulmonary permeability edema and hamper gas exchange. Tumor Necrosis Factor (TNF) is an evolutionarily conserved pleiotropic cytokine, involved in host immune defense against pathogens and cancer. TNF exists in both membrane-bound and soluble form and its mainly -but not exclusively- pro-inflammatory and cytolytic actions are mediated by partially overlapping TNFR1 and TNFR2 binding sites situated at the interface between neighboring subunits in the homo-trimer. Whereas TNFR1 signaling can mediate hyper-inflammation and impaired barrier function and AFC in the lungs, ligand stimulation of TNFR2 can protect from ventilation-induced lung injury. Spatially distinct from the TNFR binding sites, TNF harbors within its structure a lectin-like domain that rather protects lung function in ARDS. The lectin-like domain of TNF -mimicked by the 17 residue TIP peptide- represents a physiological mediator of alveolar-capillary barrier protection. and increases AFC in both hydrostatic and permeability pulmonary edema animal models. The TIP peptide directly activates the epithelial sodium channel (ENaC) -a key mediator of fluid and blood pressure control- upon binding to its α subunit, which is also a part of the non-selective cation channel (NSC). Activity of the lectin-like domain of TNF is preserved in complexes between TNF and its soluble TNFRs and can be physiologically relevant in pneumonia. Antibody- and soluble TNFR-based therapeutic strategies show considerable success in diseases such as rheumatoid arthritis, psoriasis and inflammatory bowel disease, but their chronic use can increase susceptibility to infection. Since the lectin-like domain of TNF does not interfere with TNF’s anti-bacterial actions, while exerting protective actions in the alveolar-capillary compartments, it is currently evaluated in clinical trials in ARDS and COVID-19. A more comprehensive knowledge of the precise role of the TNFR binding sites versus the lectin-like domain of TNF in lung injury, tissue hypoxia, repair and remodeling may foster the development of novel therapeutics for ARDS.

## TNF via TNF Receptors Induces Cell Death or Inflammation

About a century ago the New York surgeon William Coley observes that vaccination of cancer patients with a mixture of bacterial toxins (Coley’s toxins) induces tumor remission (Coley, 1910). Six decades later the group of Dr. Lloyd Old identifies an LPS-induced protein with profound hemorrhagic necrotic activity against tumors (mainly mediated by apoptosis in tumor-associated vasculature), as the active principle of Coley’s toxins and named it Tumor Necrosis Factor (TNF) (Carswell et al., 1975). As such, TNF was discovered at the interface between infection and cancer. Systemic treatment with high bolus doses of TNF in cancer patients is not possible, due to severe hypotension and hepatotoxicity [reviewed in Brouckaert et al. (1992)]. This problem can be partially circumvented in patients with melanoma and soft tissue sarcoma by local administration of TNF in a closed circuit, in combination with melphalan and Interferon-γ (Lejeune et al., 2006; Verhoef et al., 2007).

In the 1980s, the TNF gene is cloned by several groups in Belgium, Japan and the United States (Fransen et al., 1985; Pennica et al., 1985; Shirai et al., 1985) and is found to encode a protein that assembles into a homo-trimeric cytokine, harboring three receptor binding sites which are situated at the interface between neighboring subunits (Bodmer et al., 2002). TNF is expressed in two alternative forms (Horiuchi et al., 2010; Wajant and Beilhack, 2019) - as a surface membrane-anchored precursor, named transmembrane TNF (mTNF) (Kriegler et al., 1988), which consists of 233 residues (26 kDa), and as a soluble form (sTNF), composed of 157 amino acid residues (17 kDa), secreted upon the action of the TNF-Alpha Converting Enzyme [TACE, a.k.a A Disintegrin And Metalloprotease 17 (ADAM17)] on transmembrane TNF (Horiuchi et al., 2010; Wajant and Beilhack, 2019). Mice deficient in the natural inhibitor of TACE -Tissue Inhibitor of Metalloproteinases 3 (TIMP3)- display significantly elevated levels of TNF and severe liver inflammation (Black, 2004; Mohammed et al., 2004), highlighting the physiological importance of sTNF as a pro-inflammatory cytokine.

Tumor necrosis factor is a key mediator for the activation and recruitment of inflammatory cells, including polymorphonuclear neutrophils (PMNs) and macrophages, to sites of infection, and is as such involved in infection control, tissue repair and healing (Gardam et al., 2003). However, when generation of the cytokine is too high, this can promote organ injury.

**FIGURE 1:**
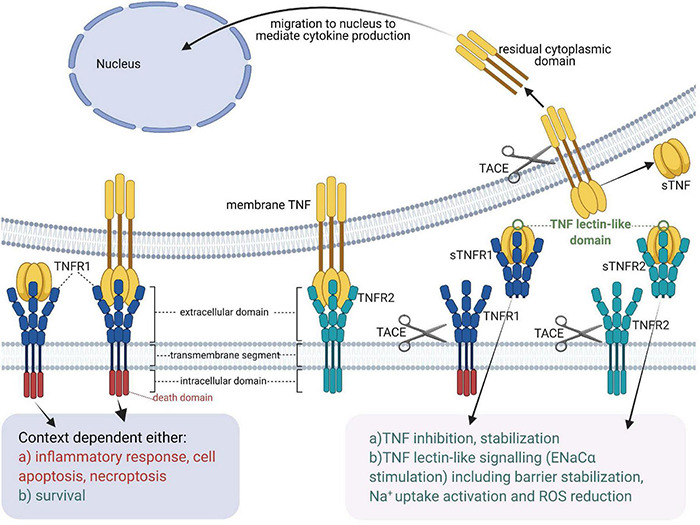
Tumor necrosis factor receptors, TNF ligands, TNF-soluble TNF receptor complexes and TNF lectin-like activity [Adapted from Horiuchi et al. (2010)]. sTNF, soluble TNF; mTNF, transmembrane TNF; sTNFR, soluble TNF receptor; TACE, TNF Alpha Converting Enzyme; TNF, tumor necrosis factor; TNFR1, membrane-associated TNF receptor 1; TNFR2, membrane-associated TNF receptor 2.

Tumor necrosis factor expression is induced upon contact with pathogen- or danger-associated molecular patterns (PAMPs and DAMPs) in immune cells, as well as in numerous types of non-immune cells, including endothelial and epithelial cells, neurons, fibroblasts, microglia and cardiomyocytes (Medler and Wajant, 2019). As summarized in Figure 1, TNF signals through two distinct membrane-associated receptors - TNF receptor 1 (TNFR1), which contains a death domain and TNF receptor 2 (TNFR2), which lacks a death domain (Wajant and Beilhack, 2019). Numerous biological functions -ranging from apoptosis and necroptosis to survival and inflammation- are mediated upon binding of TNF ligands to their receptors (Chen and Goeddel, 2002; Saperstein et al., 2009).

Whereas both transmembrane TNF (mTNF) and soluble TNF (sTNF) can bind to both TNF receptors, TNFR2, which is typically expressed in the membrane of immune cells, only becomes fully activated by transmembrane TNF (Grell et al., 1995). Both mTNF and sTNF can induce inflammatory responses (Horiuchi et al., 2010). Through cell-cell contact mTNF can also mediate juxtacrine signaling in the local medium, while sTNF launches paracrine and systemic signaling via TNFR1 even in remote locations from its source cells (Richter et al., 2012). A casein kinase I (CKI) consensus sequence in the cytoplasmic domain of mTNF was suggested to perform “reverse signaling,” which means that it can transmit signals both as a ligand and as a receptor upon interacting with TNFRs (Watts et al., 1999; Horiuchi et al., 2010).

**FIGURE 2:**
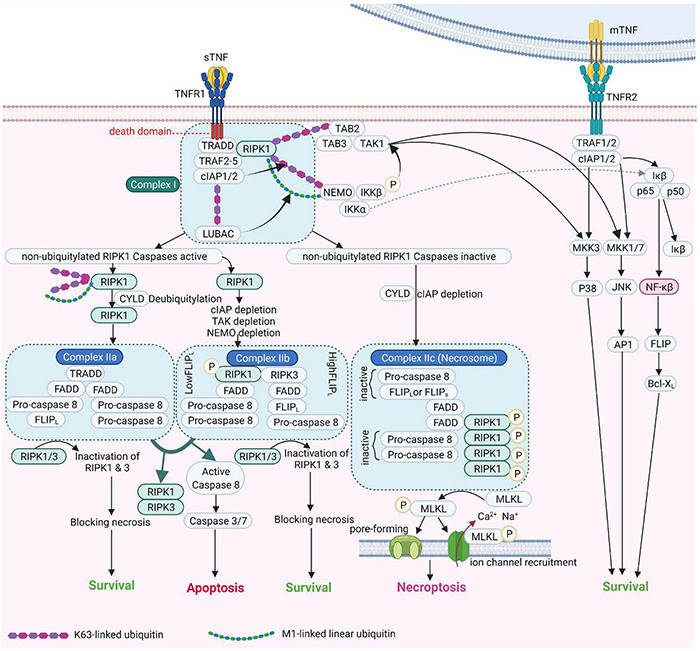
TNF Receptor-1 and TNF Receptor-2 signaling pathways [Adapted from Brenner et al. (2015) and Conrad et al. (2016)]. cIAP, Cellular Inhibitor of Apoptosis Protein; FADD, Fas-associated protein with death domain; FLIP_*L*_, FLICE-like inhibitory protein; IKK, IκB kinase; JNK, c-Jun NH2-terminal kinase; LUBAC, linear ubiquitin chain assembly complex; MLKL, mixed lineage kinase domain-like; mTNF, membrane TNF; NEMO, NF-κB essential modulator; NF-κB, Nuclear factor kappa B; NIK, NF-κB-inducing kinase; RIPK1, Receptor-interacting serine/threonine-protein kinase 1; sTNF, soluble TNF; TAK1, transforming growth factor-β Transforming growth factor beta-activated kinase 1; TNF, tumor necrosis factor; TNFR1, TNF receptor 1; TNFR2, TNF receptor 2; TRAF2, TNF receptor-associated factor 2; TRADD, TNFRSF1A Associated via death domain.

Tumor necrosis factor receptor 1 (also referred to a p55, p60, CD120a, or TNFRSF1A) (Tartaglia et al., 1991) is a prototypical, 55 kDa type 1 transmembrane glycoprotein (Tartaglia et al., 1993) with an *N*-terminal extracellular domain (ECD) comprising four cysteine-rich domains (CRD1, −2, −3, −4), an α-helical transmembrane domain, a transmembrane segment and a cytoplasmic intracellular death domain (Bodmer et al., 2002). TNFR1 is expressed by most cell types, except erythrocytes (Martínez-Reza et al., 2017). Upon binding to TNF and depending on the ubiquitination status of receptor interacting protein kinase 1 (RIPK1), TNFR1 activates several pro-inflammatory signaling pathways, including nuclear factor kappa B (NF-κB), p38 MAP kinase or JNK. Alternatively, TNFR1 induces cell death, either in the form of apoptosis or necroptosis, depending on the activity of caspase 8 (Wajant et al., 2003). These pathways are summarized in Figure 2.

Tumor necrosis factor receptor 2 (also known as p75, p80, CD120b, or TNFRSF1B) (Tartaglia et al., 1991) is a transmembrane glycoprotein with a molecular weight of 75kDa (Tartaglia et al., 1993). Similar to TNFR1, TNFR2 has an *N*-terminal ECD made by four cysteine-rich domains (CRDs) as well as an α-helical transmembrane domain. In contrast to TNFR1, the cytoplasmic domain of TNFR2 harbors no death domain (Martínez-Reza et al., 2017; Yang S. et al., 2018). TNFR2 is expressed mainly in immune cells such as regulatory T cells (Tregs), hematopoietic lineage cells, neurological tissues and endothelial cells (Richter et al., 2012; Martínez-Reza et al., 2017), some solid tumors and hematopoietic malignancies (Wajant and Beilhack, 2019). Somewhat surprisingly, TNFR2, but not TNFR1, upon interacting with mTNF or with lymphotoxin α is a crucial mediator of ICAM-1 upregulation, an adhesion molecule important in the binding of *Plasmodium*-infected erythrocytes and leukocytes to microvessels in the brain (Lucas et al., 1997b; Favre et al., 1999; Lou et al., 2001).

Although TNFR2 cannot form a death-inducing signaling complex (DISC), it nevertheless promotes TNFR1-mediated apoptosis, upon degrading anti-apoptotic factors, such as Bcl-x_*L*_ in lung microvascular endothelial cells (MVEC) (Lucas et al., 1998) or TRAF-2, which in turn prevents the binding of apoptosis inhibitory proteins to TNFR1 (Shu et al., 1996; Li et al., 2002).

Like TNF, also the TNF receptors can be cleaved from the membrane by TACE (Black, 2004). In addition, soluble TNF receptors not only neutralize, but also stabilize TNF (Aderka et al., 1992).

## The Lectin-Like Domain of TNF

Spatially distinct from the TNF receptor 1 and 2 binding sites, TNF harbors a functional domain with lectin-like activity for specific oligosaccharides, such as *N,N*′-diacetylchitobiose and branched tri-mannoses (Lucas et al., 1994). The lectin-like activity of TNF was originally discovered by its capacity to bind to uromodulin, a glycoform of Tamm-Horsfall protein expressed in the loops of Henle in the kidneys (Hession et al., 1987; Sherblom et al., 1988). Whereas TNF is able to directly kill long slender bloodstream forms of the protozoan parasite *Trypanosoma brucei brucei* causing sleeping sickness, by means of lysosomal lysis (Lucas et al., 1994; Magez et al., 1997; Daulouède et al., 2001) upon lectin-like recognition of their variant surface glycoprotein, lymphotoxin-α, which has a similar conformation as TNF and which can bind to TNF receptors, failed to do so. The domain responsible for the lectin-like activity was mapped by comparing the tertiary structures of TNF and lymphotoxin-α (Lucas et al., 1994). Moreover, soluble TNFR1, which blunts binding of TNF ligand to both TNF receptors, failed to inhibit the trypanolytic activity. The TIP peptide (a.k.a. AP301, Solnatide) is a 17 amino acid cyclic synthetic peptide (sequence CGQRETPEGAEAKPWYC) that can mimic the lectin-like domain of human TNF (Lucas et al., 1994). Mutation of the lectin-like domain of TNF does not interfere with the cytokine’s anti-bacterial activities in a cecal ligation and puncture model (Lucas et al., 1997a). There is neutralizing cross-reactivity of monoclonal antibodies raised against the lectin-like (TIP) domain of TNF and those generated against coelomic cytolytic protein 1 (CCF-1), an invertebrate analog of TNF in *Eisenia foetida*, which -like TNF- can also lyse African trypanosomes through a similar lectin-like interaction (Beschin et al., 1999, 2004). Intriguingly, although there is a high conformational similarity between both molecules, the residues making up the active site for the lectin-like activity in CCF-1 and TNF are different, as such suggesting a convergent evolution of these cytokines (Beschin et al., 1999).

## The Acute Respiratory Distress Syndrome: A Potentially Lethal Complication of Acute Lung Injury and Severe Pneumonia

The combination of predominantly type 1 and fewer tpe 2 alveolar epithelial (AT1/2) cells (make up a total surface area around 140 m^2^ in an adult human lung) and capillary endothelial cells (represent a surface area around 90 m^2^) provides a large barrier that ordinarily prevents serum proteins and fluid from leaving the capillaries and entering the alveoli. While preventing the movement of serum proteins and liquid into the alveolar space, the barriers must allow breathing, i.e. facilitate the movement of O_2_ from the alveoli into the blood and CO_2_ from the blood into the alveoli. Disruption of the alveolar epithelial barrier results in pneumonia, but additional compromise of the endothelial barrier can lead to the acute respiratory distress syndrome (ARDS) -an underdiagnosed potentially lethal complication of severe pneumonia. COVID-19, sepsis and trauma (Hariri and Hardin, 2020). The resulting accumulation of fluid, proteins and cells in the alveolar space significantly reduces breathing efficacy and patients often require mechanical ventilation. Pulmonary edema resolution is often hampered in ARDS patients and alveolar fluid clearance (AFC) capacity even inversely correlates with mortality in ARDS patients (Ware and Matthay, 2001). AFC is mediated by vectorial Na^+^ transport, with Na^+^ uptake mainly via the apically expressed epithelial sodium channel (ENaC) and Na^+^ release into the interstitial space via basolateral Na^+^-K^+^-ATPase in AT1/2 cells (Matthay et al., 2005). At least four pathological parameters must be addressed in ARDS: (i) hyperinflammation, (ii) alveolar epithelial barrier dysfunction, (iii) impaired AFC and (iv) capillary leak.

Despite recent advances in the treatment of ARDS i.e., low tidal volume ventilation (Amato et al., 1998), ventilation in prone position (Guérin et al., 2013), neuromuscular blockade (Papazian et al., 2010) and corticosteroid use (Villar et al., 2020), mortality remains as high as 30-40% (Bellani et al., 2016), at least partially due to the lack of major pharmacologic treatments for ARDS refractory to conventional therapy.

## Deleterious Outcomes of TNF-TNF Receptor Interactions in ARDS

TNF is intimately involved in inflammatory pulmonary disease, including severe pneumonia and ARDS (Mukhopadhyay et al., 2006). TNF-TNF receptor signaling has an essential impact on alveolar-capillary barrier integrity, on the physiopathology of neutrophilic alveolitis and on AFC (Lipke et al., 2010). Death signaling, AFC impairment and hyperpermeability triggered by TNF-TNFR signaling are of considerable importance in the pathogenesis of ARDS (Patel et al., 2013). Accordingly, TNF inhibitory treatments have been proposed in order to attenuate the degree of lung injury (Mazzon and Cuzzocrea, 2007).

Numerous studies have investigated the role of TNF in the regulation of capillary barrier function, but these results have to be interpreted with caution, as the reaction of cells to TNF depends on the relative expression of both TNF receptor types, the tissue, the local concentration of the ligand and its soluble TNF receptors as well as on the context, like the type of flow [laminar (high shear stress) *versus* turbulent (disturbed)] (Rangamani and Sirovich, 2007). Depending on its concentration, TNF can act either as a regulator of survival or cell death (Rangamani and Sirovich, 2007). Moreover, functional and phenotypical features of the lung microvasculature are distinct from most other vascular beds (Doerschuk, 2001) and from large pulmonary vessels, at least in part due to differential basal and TNF-induced expression of adhesion molecules (such as ICAM-1 and VCAM-1), varying requirements for leukocyte extravasation and relative susceptibility to bacterial toxins, e.g., LPS (Doerschuk, 2001). Thus, there are potential risks of erroneous extrapolation of results gained in *in vitro* studies in pulmonary large vessel endothelial cells to lung MVEC or to endothelial cells from other tissues (Bertok et al., 2011). Moreover, lung MVEC isolated from patients who developed ARDS were shown to be functionally and phenotypically different from their counterparts isolated from patients who had undergone a lobectomy for lung carcinoma. As such, constitutive expression of the adhesion molecule ICAM-1 and of TNFR2 was found to be significantly increased in pulmonary MVEC from ARDS patients, as well as their basal generation of the proinflammatory cytokine IL-6 and of the chemokine IL-8 (Grau et al., 1996). The paragraphs below provide a short overview of TNF/TNFR-mediated effects in capillary endothelial cells.

### Tumor Necrosis Factor-Induced Endothelial Cell Death and Capillary Barrier Dysfunction in the Lungs

Although TNFR1 is sufficient to induce sensitized apoptosis (in the presence of transcriptional or translational inhibitors) in primary lung mouse MVEC, the presence of both receptors is required for direct TNF-induced cell death (Lucas et al., 1998). While TNF-induced apoptosis in lung endothelium involves myosin light chain (MLC) phosphorylation, TNF-induced capillary barrier dysfunction does not (Petrache et al., 2001). Elevated plasma levels of soluble TNFRs are associated with morbidity and mortality in ALI patients (Parsons et al., 2005). Bronchoalveolar lavage fluid from early ARDS patients contains significantly elevated levels of both TNF and soluble TNF receptors, as compared to lavages from control patients investigated for chronic cough, hilar masses or pulmonary nodules (Hamacher et al., 2002).

TNF-TNFR2 interactions can be either destructive or preventive (Tartaglia et al., 1993; Lucas et al., 1997c; Wilson et al., 2007). On the one hand, TNF-TNFR2 signaling inhibits edema formation in a murine model of ventilator-induced lung injury, since mice lacking TNFR2 are more prone to the formation of edema, whereas mice not expressing TNFR1 are protected (Wilson et al., 2007). On the other hand, as indicated above, TNFR2 may increase TNFR1-mediated apoptosis upon impairing anti-apoptotic pathways (Tartaglia et al., 1993; Lucas et al., 1998; Li et al., 2002). As both TNF receptor types can mediate divergent signaling pathways, their relative expression in lung endothelial and alveolar epithelial cells can profoundly affect TNF signaling during ALI (Bertok et al., 2011).

Receptor interacting protein kinase 1(RIPK1)–dependent endothelial necroptosis underlies the TNF-induced systemic inflammatory response syndrome in mice (Zelic et al., 2018) and the blocking of connexin 43 hemichannels, which promote Ca^2+^ influx, with TAT-Gap19 protected mice against TNF-induced vascular leakage and mortality (Delvaeye et al., 2019). Laminar flow fosters pro-survival signaling, at least partially by impairing TNF-induced caspase-3 activity (Garin et al., 2007).

TNF not only decreases barrier function in ARDS by inducing cell death, but it can also affect junction protein status. Increased endothelial Ca^2+^-influx is the initial pivotal signal preceding pathways leading to contraction, including myosin light chain (MLC)-dependent mechanisms and MLC-independent microtubule rearrangement (Sandoval et al., 2001). TNF induces F-actin depolymerization in bovine pulmonary artery endothelial cell monolayers (Goldblum et al., 1993; Matsumura et al., 1997) to promote intracellular gap formation and barrier dysfunction. Across human pulmonary artery endothelial cells, TNF decreases trans-endothelial electrical resistance. This process occurs independently of MLC phosphorylation, which is catalyzed by either MLC kinase or Rho-kinase (Petrache et al., 2003). Rather, TNF causes microtubule destabilization and actin rearrangement in human pulmonary artery endothelial cells (Petrache et al., 2003). TNF-induced disassembly of the peripheral microtubule network involves activation of the p38 mitogen-activated protein kinase pathway in endothelial cells of human pulmonary arteries, which leads to an increase in permeability of endothelial cell layers (Petrache et al., 2003). Depolymerization of microtubules causes disassembly of adherens junction proteins they associate with, such as VE-cadherin, which increases permeability (Petrache et al., 2003). TNF can increase tyrosine phosphorylation of VE-cadherin (Angelini et al., 2006) and degrade the endothelial glycocalyx (Nieuwdorp et al., 2009). The Ca^2+^–dependent protein kinase C-α isoform can also play an important role in initiating endothelial cell contraction and disassembly of VE-cadherin–mediated cell–cell contacts (Petrache et al., 2003).

Tumor necrosis factor can also interact with other pathways promoting vascular leak. As such membrane-bound TNF (mTNF) and VEGF signaling pathways converge at the level of the secondary messenger, SAPK-2/p38, with continuous autocrine signaling by mTNF sensitizing endothelial cells to respond to VEGF by increasing their vascular permeability (Petrache et al., 2003).

### Tumor Necrosis Factor-Induced Oxidative and Nitrosative Stress in Pulmonary Endothelium

Although pulmonary venous and capillary endothelial cells and are at times highly exposed to O_2_-rich environments, they use the glycolytic pathway, rather than mitochondrial respiration as the main ATP-generating pathway in quiescent and proliferating states. They likely do so in order to maximize O_2_ delivery to tissues and perivascular cells. This prevents the generation of high levels of reactive oxygen species in this cell compartment (Xiao et al., 2021). The endothelium, however, also provides the first line of defense against inflammatory or infectious insults in the vasculature. Upon inflammatory stimulation by TNF, otherwise quiescent endothelial cells are activated to produce significantly increased levels of pro-inflammatory molecules and NADPH oxidase-generated ROS (Gertzberg et al., 2004), and they moreover increase PFKFB3-mediated glycolysis (Cao et al., 2019). ROS can in turn combine with nitric oxide (NO) to generate barrier-disruptive peroxynitrite (Neumann et al., 2006). Although endothelial cells were shown to express NOX-1, -2, -4 and -5, especially NOX-2 was demonstrated to contribute to TNF-induced ROS generation (Deng et al., 2012; Drummond and Sobey, 2014).

Reduction of endothelial nitric oxide synthase (eNOS)-mediated NO generation was shown to promote pulmonary microvessel leakage in eNOS^–/–^ mice (Predescu et al., 2005), suggesting a barrier-protective effect of eNOS-derived NO in the pulmonary microvasculature (Di Lorenzo et al., 2013). Increased ROS generation was shown to impair the expression and activity of eNOS (Neumann et al., 2004) and to increase the activity of arginase 1, an enzyme competing with eNOS for the common substrate arginine (Romero et al., 2008; Chandra et al., 2012; Lucas et al., 2013). TNF was shown to be critical for arginase 1 induction in a murine model of ischemia and reperfusion, since TNF^–/–^ mice had significantly reduced endothelial arginase activity (Gao et al., 2007). All of these events involve increased endothelial oxidative stress and can disrupt the vascular integrity by reducing adherens and tight junction protein expression and/or by inducing endothelial cell death. ROS can moreover increase expression of adhesion molecules in endothelium and as such foster transendothelial migration of circulating leukocytes (Wang and Doerschuk, 2000). A stop of blood flow, as can occur in TNF-induced multiple organ failure, was moreover shown to activate a mechano-sensing machinery that generates ROS and that subsequently drives neutrophil influx (Noel et al., 2013). By contrast, laminar shear stress exerts a profound anti-apoptotic effect on endothelial cells treated with TNF, via the increased NO synthesis and post-transcriptional activation of eNOS (Cicha et al., 2009).

### Tumor Necrosis Factor-Induced Upregulation of Adhesion Molecule Expression in Endothelial Cells

Apart from directly inducing barrier impairment, TNF can also indirectly induce hyperpermeability. TNFR1 activation increases neutrophil and monocyte adhesion to endothelium (Zhao et al., 2014), through upregulation of cell surface expression of endothelial cell adhesion molecules such as E-selectin, ICAM-1 and VCAM-1 in activated human pulmonary microvascular endothelial monolayers *in vitro* (Zhou et al., 2007; Su et al., 2008; Proudfoot et al., 2018). The induction of VCAM expression is associated with ROS generation, and endothelial NOX-2 is a major source of ROS associated with pulmonary inflammation (Frey et al., 2006). In patients and mice with critical limb ischemia, increased expression of the Cu^2+^ transport protein antioxidant-1 (Atox1) stimulates transcription of the NADPH oxidase organizer p47phox, thereby increasing VCAM-1/ICAM-1 expression and promoting monocyte adhesion in endothelial cells inflamed with TNF (Chen et al., 2015; Das et al., 2019).

### Tumor Necrosis Factor-Induced Release of Pro-inflammatory Markers in Endothelial Cells

The TNF-TNFR1/p38 MAPK/MK2 signaling axis induces secretion of pro-inflammatory chemokines, such as the neutrophil attractant IL-8 in human lung MVEC (Su et al., 2008). Moreover, TNF-induced activation of PI3Kγ in endothelial cells in turn induces PKC-ζ-dependent activation of NOX, leading to the generation of ROS and thereby degradation of the proteasome-dependent inhibitor of NF-κBα –IκBα- which further activates NF-κB-dependent inflammation (Frey et al., 2006).

### Tumor Necrosis Factor-Induced Thrombotic Events

TNF can promote thrombosis by inducing luminal endothelial expression of tissue factor. Tissue factor interacts with circulating coagulation factor VII and as such triggers the extrinsic coagulation pathway. This process involves NADPH oxidase activation (Clauss et al., 1996; Mechtcheriakova et al., 2001). Selective inhibitors of either TNFR1 or TNFR2-induced signaling pathways were shown to inhibit tissue factor expression (Clauss et al., 1996; Mechtcheriakova et al., 2001). However, TNFR1 is the predominant receptor responsible for the synergistic interaction between VEGF and TNF in endothelial tissue factor expression (Clauss et al., 1996; Mechtcheriakova et al., 2001).

### Tumor Necrosis Factor in COVID-19-Associated Acute Respiratory Distress Syndrome

Acute Respiratory Distress Syndrome develops in about 20% of COVID-19 patients with severe symptoms (Matthay et al., 2020; Du et al., 2021) and is influenced by age-, sex-, the presence of co-morbidities like diabetes, obesity and arterial hypertension, immunodeficiency and vaccination status. The development of COVID-19 associated ARDS is highly age-dependent and represents the main cause of COVID-19 related morbidity and mortality. As a matter of fact, COVID-19-related ARDS has increased the prevalence of ARDS in most highly industrialized countries by a factor of three (Matthay et al., 2020), leading to immense problems in terms of human resources, including excessive work load for physicians, nurses and other health care workers, with a profound impact on patients and their families, including social isolation and reduced patient care for other diseases. Severe COVID-19 is characterized by increased permeability of the alveolar-capillary barriers combined with diffuse alveolar damage, as shown in autopsies (Zhang et al., 2020). Human lung capillary endothelial cells express the main receptor for SARS-CoV2, i.e., angiotensin converting enzyme 2 (ACE-2) (Gheblawi et al., 2020), as well as of the cellular proteases, furin (Mayer et al., 2004) and TMPRSS2, necessary to mature the spike glycoprotein for subsequent receptor binding and membrane fusion (Hoffmann et al., 2020). The combination of these post-translational events causes endothelial cells to be vulnerable to SARS-CoV2. Some groups are detecting SARS-CoV2 within the pulmonary endothelium of COVID-19 patients (Ackermann et al., 2020; Varga et al., 2020) and in infected human blood vessel organoids (Monteil et al., 2020). Although therapeutic strategies to blunt host damage in COVID-19 mainly focus on the alveolar epithelium, COVID-19, particularly in the later complicated stages, is a disease of endothelial cells, particularly in the pulmonary microvasculature (Libby and Lüscher, 2020).

COVID-19-associated ARDS is accompanied by a significant imbalance in the cytokine/chemokine profile (Pedersen and Ho, 2020), with increased plasma levels of pro-inflammatory cytokines and chemokines (Pedersen and Ho, 2020). The severity of the inflammatory response, combined with T cell lymphopenia significantly contributes to pulmonary devastation and respiratory distress (Pedersen and Ho, 2020). An excessive inflammatory response during SARS-CoV-2 infection, which includes high levels of TNF, can provoke death of resident lung cells, which can further amplify barrier dysfunction. Elevated BAL and plasma levels of TNF are a hallmark manifestation of COVID-19, and can be associated with significant morbidity and mortality rates (Del Valle et al., 2020; Robinson et al., 2020). Increased soluble TNF levels can be caused by SARS-CoV2-induced activation of TACE (Haga et al., 2008; Atzeni et al., 2021). Mast cells activated by SARS-Cov-2 infection in respiratory tracts are also an important source of TNF (Kritas et al., 2020; Motta Junior et al., 2020) and together with the virus they can activate macrophages (Kritas et al., 2020). TNF is a major mediator of pulmonary edema and multi-organ failure in cases of severe COVID-19 (Eisenhut and Shin, 2020). TNF also promotes viral entry and dissemination since it induces adhesion molecule expression in endothelial cells and promotes VEGF generation, all of which contribute to increased permeability of the endothelial layer and thereby dissemination of SARS-CoV2 in patients with COVID-19 (Polidoro et al., 2020). Hyperpermeability of pulmonary blood vessels also results in improper accumulation of inflammatory monocytes, macrophages and neutrophils in the alveolar space (Channappanavar et al., 2016). Lung biopsies from patients who succumbed to severe COVID-19 show remarkable pathological injuries, with alveolar exudates, proliferation of alveolar epithelium, interstitial inflammation, and formation of hyaline membranes (Tan et al., 2020).

Activation of the coagulation pathway is another known feature of severe COVID-19 with potential development of disseminated intravascular coagulation (Teuwen et al., 2020). This is associated with pulmonary endothelial cell activation and dysfunction as cell death and the disruption of vascular endothelial integrity leads to exposure of the thrombogenic basement membrane and further in the activation of the coagulation cascade (Nachman and Rafii, 2008). In addition, IL-1β and TNF activate endothelial cells, which causes activation of coagulation cascade by expressing P-selectin, von Willebrand factor and fibrinogen, that are required for attaching platelets (Pober and Sessa, 2007).

COVID-19 endothelialitis is associated with platelet aggregation and thrombocoagulatory and fibrinolysis-inhibitory (D’Agnillo et al., 2021) features that can obstruct vessels, as manifested by the important frequency of thromboses, including pulmonary thromboembolism. This has been shown particularly in the pulmonary microvasculature, especially in vessels with a cross-sectional area between 1.25 and 5 mm^2^ (Libby and Lüscher, 2020; Morris et al., 2021), containing highly activated and functionally distinct neutrophils, which generate neutrophil extracellular traps (NETs) (Leppkes et al., 2020; Zuo et al., 2020; Panda et al., 2021), which may stimulate alternative treatment strategies besides corticosteroids and standard care (Panda et al., 2021). One has to question whether virus-associated endothelial cell injury, quite frequently co-localized with NETs, or rather the collateral of other inflammatory processes in plasma, represents the key step to neutrophil activation. The high pro-coagulation context in COVID-19 in part motivates therapeutic anticoagulation approaches especially with heparins in hospitalized patients who are not critically ill (Leppkes et al., 2020; Houston et al., 2020). Such patients seem to profit and seem less in danger of anticoagulation than sicker and frailer poly-monitorized ventilated ARDS patients, in whom both risk and benefit need to be more closely examined. To circumvent progressive respiratory failure, prone positioning of the patient, which has high preventative and therapeutic value, is recommended since it improves ventilation and perfusion mismatch (Gattinoni et al., 2020). Conceptually, the high frequency of the above-mentioned pulmonary embolism, including the site of peripheral arterial large vessel occlusions like coronary, cerebral, limb, and other arterial occlusions, may have a similar pathogenetic background, which may also be the case for COVID-19 pneumonia and makes it a more privileged respiratory disease, as compared to non-COVID ARDS in terms of prognosis.

### Tumor Necrosis Factor Neutralizing Agents

**FIGURE 3:**
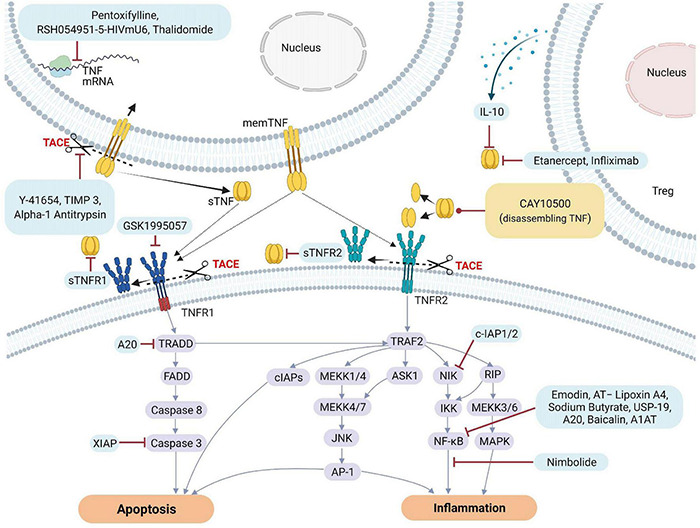
Natural and pharmacological inhibitors of TNF and TNF receptor expression and bioactivity. AP-1, Activator protein 1; AQP5, Aquaporin 5; ASK1, Apoptosis signal-regulating kinase 1; ASM, acid sphingomyelinase; cIAP1, Cellular Inhibitor of Apoptosis Protein 1; ENaC, epithelial sodium channel; FADD, Fas-associated protein with death domain; ICAM-1, intercellular adhesion molecule-1; IL, Interleukin; JNK, c-Jun NH2-terminal kinase; MAPK, mitogen-activated protein kinase; MEKK, mitogen-activated protein Kinase/ERK kinase kinase; mRNA, messenger RNA; NF-κB, Nuclear factor kappa B; NIK, NF-κB-inducing kinase; sTNF, soluble TNF; TACE, TNF converting enzyme; TJs, tight junctions; TNF, tumor necrosis factor; TNFR1, TNF receptor 1; TNFR2, TNF receptor 2; TRAF2, TNF receptor-associated factor 2; TRADD, TNFRSF1A Associated via death domain; XIAP, X-Linked inhibitor of apoptosis.

To counteract excessive deleterious TNF signaling leading to tissue damage, barrier dysfunction and hyperinflammation, several types of molecular-based therapeutic approaches have been developed over the past decades (Fischer et al., 2020). These include monoclonal antibodies such as infliximab (Remicade), adalimumab (Humira), certolizumab pegol (Cimzia), golimumab (Simponi) and soluble TNF receptor constructs, such as etanercept (Enbrel) (Russell et al., 2020). TNF inhibitors may directly compete with the TNF ligand, or deactivate its receptors or the enzyme generating the soluble TNF ligand, i.e., TACE, resulting in inhibition of downstream signaling pathways leading to cell death or inflammation. Figure 3 illustrates some of the different targets associated with TNF and TNFRs that can be inhibited by natural or pharmacological TNF blockers. In addition, data obtained from various studies and clinical trials on the impact of natural or pharmaceutically designed TNF blockers are summarized in Tables 1A,B.

**TABLE 1A:** Impact of TNF inhibitors/stimulators in experimental settings using non-human models including non-human primates (NHP) and non-primates mammals (NPM)

Effective agent (structure)	Experimental model	Proposed mechanism of action (outcome)	References
**Alpha 1 -antitrypsin (A1AT)** (glycosylated protein)	*In vitro*: Human lung microvascular endothelial cells	**Native and oxidized forms of A1AT show inhibitory effects on TNF stimulated gene expression** (A1AT limits the uncontrolled endothelial cell activation and vascular injury in inflammatory disease)	Subramaniyam et al., 2008
**A20/TNFAIP3** (deubiquitinating enzymes)	*In vivo*: NPM(rat)	**Inhibition of TNF induced NF-kB p65 expression** (Alleviation of pulmonary inflammation)	Wu et al., 2017
**Anti-TNF antibody** (polyclonal anti-TNF antibody)	*In vivo*; NPM(Rabbits)	**Inactivation of TNF by binding to TNF molecules** (Improvement in oxygenation and respiratory compliance, ↓ infiltration of leukocytes, attenuation in lung injury)	Imai et al., 1999
**Anti-TNF antibody** (rabbit anti-TNF antibody)	*In vitro*: Wistar rats lung epithelial cells	**Inactivating TNF by binding to TNF molecules** (↓ alveolar epithelial permeability)	Li et al., 1995
**Anti-TNF monoclonal antibody** (murine IgG1 antibody)	*In vivo*: NPM(Porcine)	**Inactivating TNF by binding to TNF molecules** (Suppression of systemic hemodynamic actions of TNF, ↓ PMN adhesion receptors - > inhibiting PMN/endothelial cell interaction)	Windsor et al., 1993
**Anti-TNF antibody** (monoclonal rat anti mouse antibody)	*In vitro*: L929 fibrosarcoma cell line PU	**Deactivating TNF by binding to TNF molecules** I(Inhibiting TNF-mediated activities such as Tumouricidal activity of activated macrophages)	Lucas et al., 1990
**Anti-TNF antibody** (polyclonal rabbit anti-murine-TNF antiserum)	*In vivo*: NPM(Sprague-Dawley rats)	**Neutralizing both recombinant and natural TNF** (Amelioration of pulmonary microvascular permeability, no prevention of pulmonary neutrophil sequestration)	Caty et al., 1990
**TIP peptide (AP301/Solnatide** (lectin-like domain of TNF) **(mutant of TIP peptide with increased ENaC-activating effect)**	*In vitro*: Human alveolar epithelial A549 cells & human embryonic kidney HEK-293 cells	**Interaction with ENaC** (Solnatide restored function in ENaC-α frameshift mutants to current density levels of wild type ENaC or higher)	Willam et al., 2017a,b
	*In vitro*: Human alveolar epithelial A549 cells & human embryonic kidney	**Stimulation of the TNF lectin-like region** (Normalization of both sodium and fluid absorption in oedematous alveoli to non-oedematous levels through an N-glycan dependent interaction between the TIP-peptide and ENaC)	Shabbir et al., 2015
	*In vivo*: NPM(C57BL/6 mice infected with pneumonia virus)	**No effect** (TIP did not affect pulmonary edema nor course of disease)	van den Berg et al., 2014
	*-In vitro*: rat alveolar type II cells *-In vivo*: NPM (dog & pig)	**Interaction with ENaC** (↑ amiloride-sensitive Na^+^ current in primary dog, pig and rat alveolar type II cells)	Tzotzos et al., 2013
	*In vivo*: NPM (Sprague-Dawley rats)	**Stimulation of the TNF lectin- like region** (↓ pulmonary edema, ↑ occludin expression, improved gas-blood barrier function)	Zhou et al., 2017
**AT-Lipoxin A4** (an endogenous lipoxygenase-derived eicosanoid mediator)	*In vivo*: NPM(rat)	**Inhibition of NF-κB** (Attenuation of the expression of TNFR1, TRADD, TRAF2 & RIP; inhibition of inflammatory mediator generation in severe acute pancreatitis-associated lung injury)	Yu et al., 2019
**Atorvastatin** (C33H35FN2O5)	*In vivo*: NPM(mice)	**Reducing TNF level** (Atorvastatin modulates inflammation via a reduction in TNF, redox markers including superoxide dismutase and catalase, & lipid peroxidation agents such as malondialdehyde and hydroperoxides that play a key role in ALI development)	Melo et al., 2013
**Baicalin** (5,6-dihydroxy-7-O-glucuronideflavone, BA)	*In vivo*: NPM(Kunming mice)	**Inhibiting TLR4/JNK/ERK/NF-κB Pathway** ↓(Downregulation of proinflammatory factors such as TNF, IL-1β & total proteins in BALF; alleviation of the lung injury score)	Long et al., 2020
**BMS-470539** (agonist of melanocortin 1 receptor)	*-In vitro*: Mouse neutrophils *-In vivo*: NPM(Balb/c mice)	**Suppression of TNF production** (Inhibition of proinflammatory responses; suppressing neutrophil infiltration and protecting tissue, improving lipopolysaccharide-induced ALI and mortality in mice)	Jang et al., 2019
**β-glucan** (a principal component of fungal cell walls)	*In vitro*: rabbit alveolar macrophages	**Stimulation/Suppression of TNF production** (Concentration dependent stimulation (100 to 200 mg) and suppression (>500 mg/ml β-glucan) of the released TNF from macrophages)	Olson et al., 1996
**Dexamethasone** (C22H29FO5)	*In vivo*: NPM(Sprague-Dawley rats)	**Blocking TNF binding to TNFR1 by TNF subunit disassembling** (↓ TNF in plasma, no change in the TNF levels in the lung tissue, lightly inhibition of leukocyte recruitment and infiltration into the lung, no -reduction in lung edema and no improvement in the lung function)	Moreira et al., 1993
**Dexamethasone** (C22H29FO5)	*In vitro*: Human monocytes	**Inhibition of TNF with not defined mechanisms** (↓ TNF production, inhibits TNF production primarily posttranscriptionally by reducing translation)	Moreira et al., 1993
**Emodin** (6-methyl-1,3,8-trihydroxyanthraquinone)	*In vivo*: NPM(C57/black mice)	**Inhibition of NF-κB** (↓ pulmonary inflammation in ARDS)	Liu et al., 2020
**Etanercept** recombinant protein constructed by fusing human extracellular domain of sTNFR2 to human IgG1; TNFR-Fc)	*In vivo*: NPM(BALB/c mice)	**Inactivation of TNF by binding to TNF molecules** (↓ proinflammatory cytokines; ↓ pulmonary vascular leakage, preservation of lung parenchymal architecture, interruption in inflammation-oxidative stress feedback by inhibiting kinase activation and NF-κB nuclear translocation, inhibiting damage to the lung tissue)	Weifeng et al., 2016
	*In vivo*: NPM(C57BL6 mice*in situ/ex vivo*)	**Inactivation of TNF by binding to TNF molecules** (↓ NF-κB activation during ischemic acute kidney injury, ↓ lung microvascular barrier dysfunction, ↑ alveolar liquid clearance)	White et al., 2012
**Infliximab** (monoclonal anti-human TNF antibody; IgG1κ mAb)	*In vivo*: NPM(mice)	**Inactivation of TNF by binding to TNF molecules** (attenuation in the development of TJs alteration in acute inflammation)	Braun et al., 2005
	*In situ*: NPM(Wistar rats)	**Inhibition of sTNF** (↑ alveolar liquid clearance)	Braun et al., 2005
**InVivoMab, BE0058** (monoclonal anti-TNF antibody)	*In vivo*: NPM(BALB/c mice)	**Inactivating of TNF by binding to TNF molecules** (↓ TNF in BALF, no effect on IL-1α and IL-6, ↓ neutrophil infiltration)	Gu et al., 2020
**MAb** (anti-TNF monoclonal antibody)	*In vivo*: NPM(Sprague-Dawley rats)	**Inhibition of TNF** (Intestinal ischemia-reperfusion in rats, which leads to stimulation of alveolar liquid clearance and that this stimulation is mediated at least in part, by a TNF-dependent mechanism)	Börjesson et al., 2000
**Monoclonal anti-mouse TNF antibody**	*In vivo*: NPM(Rats)	**Inhibition of TNF** (Inhibition of the increase in alveolar fluid clearance that occurs 24 hours after instillation of bacteria into the distal air spaces of rat lung)	Rezaiguia et al., 1997
**Monoclonal neutralizing anti-rat TNF antibody**	*In vivo*: NPM(rats)	**Inhibition of sTNF** (TNF-dependent increase in alveolar fluid movement in a model of severe bronchial allergic inflammation associated with endothelial and epithelial leakage is observed)	Tillie-Leblond et al., 2002
**Nimbolide** (chemical constituent of *Azadirachta indica*)	-*In vitro*: murine macrophages -*In vitro*: human type II alveolar epithelial cells	**Inhibition of TNF mediated NF-κB and HDAC-3 nuclear translocation** (↓ inflammatory cytokines and maintaining redox balance; alleviation of the inflammatory symptoms associated with ARDS; ↓ mitochondrial ROS)	Pooladanda et al., 2019
**Pentoxifylline** (C13H18N4O3)	*In vitro*: Human monocytes	**Suppression of TNF mRNA expression** (Inhibition of TNF and GM-CSF production)	Moreira et al., 1993
**Pravastatin** (a 3-hydroxy-3-methylglutaryl coenzyme A)	*In vivo*: NPM (BALB/c mice)	**Decreasing TNF level** (Pravastatin shows protective effect against LPS-induced lung vascular leak and inflammation in murine lipopolysaccharide-induced ALI)	Yao et al., 2006
**Rosuvastatin** (3-hydroxy-methyl-3-glutamyl coenzyme A reductase inhibitor)	*In vivo*: NPM(Rats)	**Reduction intrapulmonary shunts and plasma levels of vascular endothelial growth factor and TNF** (Rosuvastatin alleviates experimental hepatopulmonary syndrome through blockade of pulmonary inflammatory angiogenesis via TNF/NF-κB and VEGF/Rho-associated A kinase pathways down-regulation)	Chang et al., 2015
**RSH054951-5-HIVmU6** (Short hairpin RNA)	*In vivo*: NPM(Sprague-Dawley rats)	**Inhibition of TNF expression by targeting TNF mRNA** (Alleviation of ALI, ↑ IL-10 expression in lung tissues)	Yang Z. et al., 2018
**Sodium butyrate** (natural short chain 4-carbon fatty acid salt in animal fats)	*In vivo*: NPM(Balb/c mice)	**Inhibition of NF-κB** (Inhibition of TNF & IL-6 production and alleviates LPS-induced ALI in mice)	Li et al., 2018
**Simvastatin** (hydroxymethylglutaryl-coenzyme A reductase inhibitor; a lipid-lowering medication)	*In vivo*: NPM(BALB/c mice)	**Decreasing in IL-4, IL-13, and TNF concentration in lung lavage fluid** (Attenuation of allergic airway inflammation, inhibition of key helper T cell type 1 and 2 chemokines, and improvement of lung physiology in a mouse model of asthma)	Zeki et al., 2009
**sTNFR** (Human recombinant sTNFRs)	*In vivo*: NPM(Sprague-Dawley rats)	**Inhibition of TNF from binding to the TNFRs** (Preventing bowel ischemia-induced lung neutrophil sequestration, preventing TNF-mediated lung injury after intestinal ischemia)	Sorkine et al., 1995
**Thalidomide** (α-N-phthalimidoglutarimide)	*In vitro*: Human monocytes	**Increasing degradation of TNF mRNA** (Inhibition of TNF release from LPS-stimulated monocytes in a concentration-dependent fashion; suppression of TNF mRNA expression)	Moreira et al., 1993
**TIMP-3** (tissue inhibitors of metalloproteinases 3)	*In vitro*: myeloma cells of mouse	**Inhibition of TACE**	Amour et al., 1998
**TN3-19.12** (hamster anti-mouse TNF monoclonal antibody, IgG fragment)	*In vivo*: NPM (C57BL6 mice)	**Inhibition of TNFR1** (p55-targeting dAb attenuates lung injury and edema formation in mice model of ARDS induced by acid aspiration, with protection with a single dose lasting up to 24 h)	Wilson et al., 2017
	*In vivo*: NHP(C57BL6 mice)	**Inhibition of TNFR1 signaling** (Inhibition of TNF-mediated cell cytotoxic activity, I.V.: ↓ neutrophil recruitment, ameliorates pulmonary edema and inflammation during VILI)	Bertok et al., 2012
	*In vivo*: NHP(C57BL6 mice)	**Inhibition of TNFR1 signaling** (I.T.: ↓ neutrophilic alveolitis in VILI & ↓ pulmonary inflammation, I.V.: no reduction in neutrophil infiltration)	Wilson et al., 2005
**USP19** (deubiquitinating enzymes)	*In vivo*: NPM(mice)	**Inhibition of NF-κB & MAPK by deubiquitinating TAK1** (Inhibition the production of proinflammatory mediators and activation of TNF downstream signals)	Lei et al., 2019
**Y-41654** low-molecular-weight TACE inhibitor)	*In vitro*: alveolar macrophages of Sprague-Dawley rats	**Inhibition of TACE** (↓ sTNF concentration)	Shimizu et al., 2009
	*In vivo*: NHP(Sprague-Dawley rats)	**Inhibition of TACE** (↓ concentrations of sTNF and proteins in BALF, ↓ number of neutrophils in lung tissue and BALF)	Shimizu et al., 2009
	*In vivo*: NHP(Lewis rats)	**Inhibition of TACE** (↓ neutrophil accumulation in the alveolar space, ↓ ICAM-1, ↓ MCP-1, ↓ CINC, ↓ HMGB1, and ↓ soluble epithelial cadherin and ↓ neutrophil elastase activity in BALF, ↓ endothelial and alveolar septal damage, ↓ transplantation-related ALI)	Goto et al., 2004

*↑, upregulation/increase; ↓, downregulation/decrease; A1AT, Alpha 1-antitrypsin; ALI, acute lung injury; ARDS, acute respiratory distress syndrome; BALF, bronchoalveolar lavage fluids; BW, Body weight; CINC, Cytokine-induced neutrophil chemoattractant; GM-CSF, Granulocyte-macrophage colony-stimulating factor; HMGB1, High mobility group box 1; ICAM-1, intercellular adhesion molecule-1; I.T., intratracheally; I.V., intravenously; κ mAb, kappa Monoclonal Antibody; MCP-1, monocyte chemotactic protein-1; mRNA, messenger RNA; PMN, polymorphonuclear leukocytes; ROS, reactive oxygen species; sTNF, soluble TNF; TACE, TNF converting enzyme; TAK1, TGF-β-activated kinase 1; TJs, tight junctions; TNFR1, TNF receptor 1; VCAM-1, vascular cell adhesion molecule-1; VILI, ventilator-induced lung injury.*

**TABLE 1B:** Impact of TNF inhibitor/stimulators or lectin-like domain stimulators in experimental settings using human trials with diseased human beings (DH) or volunteer human beings (H).

Effective agent (structure)	Experimental model	Experimental design in human studies	Proposed mechanism of action (outcome)	References
**TIP peptide (AP301/Solnatide** (lectin-like domain of TNF)	*In vivo*: DH (patients with moderate-to-severe ARDS)	Randomized to parallel groups receiving escalating doses of Solnatide or placebo, respectively. *n* = 30, 10 trial and 20 placebo)	**TNF lectin-like region** (Identification of safe doses of Solnatide for inhalative administration in ARDS patients have been discussed, thus no formal primary and secondary outcomes are defined)	Schmid et al., 2021
	*In vivo*: DH (patients with primary graft dysfunction after lung transplantation)	Randomized, placebo-controlled, single-center pilot-study; *n* = 20	**TNF lectin-like region** (It caused improve in gas exchange led to a significantly shorter duration of mechanical ventilation and a trend towards a shorter ICU stay)	Aigner et al., 2017
	*In vivo*: DH (in mechanically ventilated patients with ARDS)	Single-center, randomized, double-blind, placebo-controlled clinical trial; *n* = 20	**TNF lectin-like region** (Results shows reduced EVLWI in patients with SOFA scores ≥11 receiving AP301)	Krenn et al., 2017
	*In vivo*: DH (mechanically ventilated ICU patients)	Randomized, double-blind, placebo-controlled, parallel-group; *n* = 40	**TNF lectin-like region** (Oral inhalation of AP301 peptide activates pulmonary edema clearance)	Krenn et al., 2014
	*In vivo*: H	Randomized, double blind, placebo-controlled, parallel group, sequential ascending design *n* = 48	**TNF lectin-like region** (Inhaled AP301 single doses up to 120 mg were safe and well tolerated. Distribution of AP301 was largely confined to the lung)	Schwameis et al., 2014
**Afelimomab** (anti-TNF antibody)	*In vivo: DH*(patients with severe sepsis syndrome)	Prospective, randomized, double-blind, placebo-controlled, multiple-center, phase III clinical trial; *n* = 998	**Inhibition of TNF** (↓ TNF level in blood; Afelimomab is safe, biologically active, and well tolerated in patients with severe sepsis, reduces 28-day all-cause mortality, and attenuates the severity of organ dysfunction in patients with elevated interleukin-6 levels)	Panacek et al., 2004
	*In vivo: DH*(patients with sepsis syndrome)	Multicenter, open-label, prospective, randomized, dose-ranging pharmacokinetic study; *n* = 48	**Inhibition of TNF** (Multidose therapy with Afelimomab was safe, well tolerated, and had predictable linear kinetics)	Gallagher et al., 2001
	*In vivo: DH*(patients with severe sepsis syndrome)	Multicentre, double-blind, randomized, placebo-controlled study; *n* = 446	**Inhibition of TNF** (A small (3.7%) absolute reduction in mortality rate was found in the Afelimomab-treated patients. The treatment difference did not reach statistical significance)	Reinhart et al., 2001
**Atorvastatin** (C33H35FN2O5)	*In vivo*: DH (Patients with aspiration pneumonia complicated with cerebral infarction)	Controlled clinical trial; *n* = 160	**Decreasing the level of IL-6, IL-8 and TNF in blood** (Atorvastatin is effective in the treatment of cerebral infarction patients complicated with aspiration pneumonia)	Wei and Liu, 2018
**AZD9773** (an ovine, polyclonal, anti-human TNF Fab preparation)	*In vivo*: DH (patients with severe sepsis)	Phase II, double-blind, placebo-controlled, dose-escalation study	**Inhibition of TNF** (AZD9773 was generally well tolerated with dose-proportional pharmacokinetics in Japanese patients with severe sepsis/septic shock)	Aikawa et al., 2013
	*In vivo*: DH (patients with severe sepsis)	Double-blind, placebo-controlled, multicenter Phase IIa study	**Inhibition of TNF** (↓ TNF level in blood; The safety, pharmacokinetic and pharmacodynamics parameters data support the continued evaluation of AZD9773 in larger Phase IIb/III studies)	Morris et al., 2012
**BAYx1351** (Anti-TNF antibody, murine monoclonal antibody)	*In vivo*: DH (patients admitted to the hospital for shock due to sepsis)	Randomized, multicenter, double-blind, placebo-controlled clinical trial in 105 hospitals	**Inactivating TNF by binding to TNF molecules** (No improvement in survival after septic shock)	Abraham et al., 1998
**BAYx1351** (monoclonal antibody to human TNF)	*In vivo*: DH (patients with sepsis syndrome)	Randomized, controlled, double-blind, multicenter clinical trial; *n* = 971	**Inhibition of TNF** (In septic shock patients who received TNF MAb, a significant reduction in mortality in 3 days after infusion occurred, the trend toward reduced mortality continued at 28 days following treatment, the difference in mortality among shock patients treated with placebo or TNF- MAb was not significant)	Abraham et al., 1995
**BAY x 1351** (murine monoclonal antibody to recombinant human)	*In vivo: DH*(patients with sepsis syndrome)	International, multicentre, prospective, placebo-controlled trial in patients with sepsis; *n* = 553	**Inhibition of TNF** (↓ TNF level in blood; The study suggested a possible role for anti-TNF antibody as adjunctive therapy)	Cohen and Carlet, 1996
**CA2** (a chimeric neutralizing antibody to TNF)	*In vivo: DH*(patients with severe sepsis syndrome)	Randomized, double-blind, placebo-controlled trial; *n* = 56	**Inhibition of TNF** (A single dose of cA2 did not alter the overall pattern of cytokine activation or the profound derangements in physiologic function that accompany severe sepsis)	Clark et al., 1998
**CB006** (monoclonal antibody to human TNF)	*In vivo: DH*(patients with sepsis syndrome)	Open-label, prospective, phase II multicentre (12 Twelve academic medical center) trial with escalating doses	**Inhibition of TNF** (CB0006 has proven to be safe in this clinical trial and may prove to be useful in septic patients with increased circulating TNF concentrations)	Fisher et al., 1993
**CDP571** (fully humanized anti-TNF antibody)	*In vivo*: DH (patients with septic shock)	Prospective, randomized, placebo-controlled, phase II multicenter clinical trial, with escalating doses CDP571; *n* = 42	**Inhibition of TNF** (CDP571, is well tolerated and able to cause a dose-dependent reduction in circulating TNF concentrations in patients with septic shock)	Dhainaut et al., 1995
**CytoFab** (polyclonal ovine anti-TNF fragment antigen binding fragments)	*In vivo: DH*(patients with severe sepsis)	Phase II, randomized, blinded, placebo-controlled trial in 19 intensive care units; *n* = 81	**Inhibition of TNF** (CytoFab is well tolerated in patients with severe sepsis, effectively reducing serum and BAL TNF and serum IL-6 concentrations and increasing the number of ventilator-free and ICU-free days at day 28)	Rice et al., 2006
**GSK2862277** (anti tTNFR1 domain antibody)	*In vivo*: DH (patients undergoing elective transthoracic oesophagectomy)	Randomized, placebo-controlled pilot study; *n* = 33	**Antagonizing TNFR1** (lower postoperative alveolar capillary leak or extra vascular lung water, less postoperative lung injury than historically reported)	Ryan et al., 2020
**Lenercept TNFsr-p55** (p55 tumor necrosis factor receptor fusion protein)	*In vivo: DH*(patients with severe sepsis syndrome)	Randomized, double blind, placebo-controlled, multicenter phase III trial; *n* = 1342	**Inhibition of TNF** (Lenercept had no significant effect on mortality in the study population)	Abraham et al., 2001
**MAK195F** (anti TNF antibody fragment)	*In vivo: DH*(patients with sepsis or septic shock)	Prospective, randomized, open label, placebo-controlled, dose-ranging, multicentre (16 academic medical centres), multinational clinical trial; *n* = 122	**Inhibition of TNF** (There was no increase in survival from sepsis for the patients receiving anti-TNF treatment in the overall study population)	Reinhart et al., 1996
**p55-IgG** (anti TNFR1 domain antibody)	*In vivo*: DH (patients with refractory shock or severe sepsis)	Randomized controlled multicenter trial; *n* = 201	**Antagonizing TNFR1** (No decrease in mortality between placebo and p55-IgG in all infused patients; patients with severe sepsis who received p55-IgG, there was a trend toward reduced mortality at day 28)	Abraham et al., 1997
**Rosuvastatin** (C_22_H_28_FN_3_O_6_S)	*In vivo*: DH (patients with stable COPD)	Controlled clinical trial; *n* = 110	**Decreasing TNF level in blood** (Rosuvastatin reduces systemic inflammation in COPD patients)	Samorukova et al., 2017
**Simvastatin** (hydroxymethylglutaryl-coenzyme A reductase inhibitor; a lipid-lowering medication)	*In vivo*: H	Randomized, double-blind, placebo-controlled clinical trial	**Inhibition of TNF with not defined mechanisms** (Simvastatin reduced the release of TNF induced by LPS)	Shyamsundar et al., 2009
**TNFsr-p75** (recombinant, soluble fusion protein that is a dimer of an extracellular portion of the human TNFR and the Fc portion of IgG1)	*In vivo*: DH (patients with septic shock)	Randomized, double blind, placebo-controlled, multicenter trial. *N* = 141	**Inhibition of TNF** (Treatment with the TNFR:Fc fusion protein does not reduce mortality, and higher doses appear to be associated with increased mortality)	Fisher et al., 1996

*↑, upregulation/increase; ↓, downregulation/decrease; ALI, acute lung injury; ARDS, acute respiratory distress syndrome; BALF, bronchoalveolar lavage fluids; BW, Body weight; ICAM-1, intercellular adhesion molecule-1; MCP-1, monocyte chemotactic protein-1; mRNA, messenger RNA; PMN, polymorphonuclear leukocytes; ROS, reactive oxygen species; subq., subcutaneously; sTNF, soluble TNF; TACE, TNF converting enzyme; TNFR1, TNF receptor 1; VCAM-1, vascular cell adhesion molecule-1; VILI, ventilator-induced lung injury.*

Despite the involvement of a vast variety of pro-inflammatory mediators, exclusive blockage of TNF seems to result in better clinical outcomes in a broad range of diseases associated with inflammation and autoimmunity and clinical trials assessing TNF inhibitors for treatment of ARDS in severe COVID-19 patients have been proposed (Feldmann et al., 2020). Drugs with anti-inflammatory and/or anti-viral effects can be considered as add-on therapy against acute lung injury- (Gallelli et al., 2020) and COVID-19-associated hyper-inflammation (Robinson et al., 2020). Indeed, genetic or pharmacological inhibition of TNF and TNFRs abrogates production of TNF in pleural exudates and lung tissues and reduces infiltration of inflammatory cells into the pleural cavity and lung tissues, alveolar-capillary barrier dysfunction and resident lung cell apoptosis (Mazzon and Cuzzocrea, 2007). TNF neutralization has shown protection against SARS-CoV2 infection in animal models (Jamilloux et al., 2020).

As a note of caution, the observation of higher rates of, sometimes fatal, bacterial infection in patients with rheumatoid arthritis and psoriasis treated with TNF neutralizing antibodies and soluble receptors (Keane et al., 2001) clearly documents the danger of chronically suppressing these sometimes crucial bactericidal pathways. Taken together, these findings demonstrate limitations of chronically using TNF neutralizing agents that blunt binding of the ligand to its receptors, since this approach can inhibit both deleterious and beneficial TNF-TNFR signaling. The search for alternative treatments that selectively impair TNF-mediated vascular dysfunction, AFC impairment and hyper-inflammation in cells of the alveolar-capillary compartment, without impairing the cytokine’s beneficial actions in host response to infection, is therefore therapeutically significant.

## The Yin and Yang of the Tumor Necrosis Factor Molecule in the Lungs – the Lectin-Like Domain *Versus* the Receptor Binding Sites of Tumor Necrosis Factor

### Divergent Roles of Tumor Necrosis Factor’s Receptor Binding Sites and Lectin-Like Domain in Alveolar Fluid Clearance

Alveolar fluid clearance is essential to keep the alveolar space relatively dry, to assure efficient gas exchange. AFC is mediated by vectorial Na^+^ transport through the apically expressed epithelial sodium channel (ENaC) and basolateral Na^+^-K^+^-ATPase in type I and II alveolar epithelial cells (Matthay et al., 2005; Vadász et al., 2007). The amiloride-sensitive ENaC in its native configuration consists of three subunits of α, β, and γ and controls the overall rate of transapical Na^+^ transport (Dagenais et al., 2004). A fourth δ subunit was also identified and can replace the α subunit to form an alternative active ENaC configuration (Ji et al., 2012). Na^+^ absorption by ENaC is the critical driving force of AFC at birth and is crucially involved in pulmonary edema clearance in adulthood (Dagenais et al., 2004). Hence, mice lacking ENaC-α die from alveolar flooding at birth (Hummler et al., 1996).

Epithelial sodium channel activity is regulated by subunit maturation upon cleavage of the extracellular loops of the α and γ subunits by proteases such as furin and prostasin (Kleyman and Eaton, 2020), by its cell surface expression *N* and by its open probability time *Po*, the latter of which is at least partially controlled by complex formation of ENaC with the protein MARCKS and with PIP_2_ (Alli et al., 2012, 2015; Archer et al., 2020).

Conflicting study outcomes are found with regard to the effect of TNF on AFC. Whereas TNF generation is increased in lavage from acute ARDS patients (Hamacher et al., 2002), whose AFC capacity inversely correlates with mortality (Ware and Matthay, 2001), a TNF-dependent increase in AFC is found in a number of animal models, three of which are presented in more detail (Rezaiguia et al., 1997; Börjesson et al., 2000; Tillie-Leblond et al., 2002; Hamacher et al., 2018). Further studies show that TNF can either stimulate (Fukuda et al., 2001) or inhibit ENaC in alveolar epithelial cells (Johnson et al., 2002, 2006; Dagenais et al., 2004; Eaton et al., 2004) and that, accordingly, the cytokine has a substantial impact on the capacity of alveolar epithelial cells to transport Na^+^ (Dagenais et al., 2004). In the following paragraphs we will discuss the opposing actions of TNF-TNFR1 signaling and activities of the lectin-like domain of TNF on ENaC.

The inhibitory effect of TNF on ENaC is mainly mediated by TNFR1 (Dagenais et al., 2004; Braun et al., 2005). TNF decreases the mRNA expression levels of α, β, and γ subunits of ENaC in alveolar epithelial cells isolated from male Sprague-Dawley rats (Dagenais et al., 2004), which correlates with a significant reduction in amiloride-sensitive current in these cells (Dagenais et al., 2004). TNF moreover affects the stability of ENaC mRNA (Dagenais et al., 2004). In addition, TNF downregulates ENaC-α mRNA involving a complex process that requires the transcription of multiple genes (Dagenais et al., 2004). Along with the abovementioned results, direct exposure of rat type II alveolar epithelial cells to TNF inhibits mRNA and protein expression of the α- and γ-, but not of the β subunit of ENaC (Yamagata et al., 2009) and impairs ENaC function, as revealed by reduced amiloride-sensitive currents in these cells (Yamagata et al., 2009). These results document the substantial negative influence TNF can exert on the capacity of alveolar epithelial cells to transport sodium (Dagenais et al., 2004).

One of the mechanisms through which TNF can impair ENaC activity is through the TNFR1-mediated activation of acid sphingomyelinase (ASMase), an enzyme located in lysosomes and in the extracellular space, which catalyzes the conversion of sphingomyelin, a major component of membranes, into ceramide and phosphocholine (Gorelik et al., 2016). Low levels of ASMase maintain an equilibrium within the alveolar space between ceramides and sphingomyelin substrate (Ryan et al., 2003), but during inflammation this balance can be altered, resulting in increased generation of injurious sphingolipids, that can in turn accelerate pulmonary edema formation (Schissel et al., 1996; Haimovitz-Friedman et al., 1997; Ryan et al., 2003). Ceramide is a crucial mediator of TNF/TNFR1-induced inhibition of ENaC, via a pathway associated with PKC-dependent externalization of phosphatidyl serine (Bao et al., 2007).

Apart from inhibiting ENaC, TNF/TNFR1 signaling can also affect mRNA and/or protein expression of other channels involved in AFC. As such, TNF impairs expression of the major water channel aquaporin 5, expressed in alveolar, tracheal, and upper bronchial epithelium, through a mechanism involving activated NF-κB (Towne et al., 2000, 2001). Moreover, TNF downregulates the steady-state mRNA level of the cystic fibrosis transmembrane conductance regulator (CFTR) in HT-29 cells (Nakamura et al., 1992). Depending on the direction of the chloride transport, CFTR can either activate (inward) or inhibit (outward) ENaC activity. Similar to the impact of TNF on ENaC, its inhibitory action on CFTR occurs at the post-transcriptional level in a dose- and time-dependent manner (Nakamura et al., 1992), by reducing CFTR mRNA half-life (Nakamura et al., 1992).

In a *Pseudomonas aeruginosa* (*P. aeruginosa*)-induced pneumonia model in rats, Rezaiguia et al., measured alveolar protein and liquid clearance, 24 h after intratracheal instillation of bacteria, by instilling 5% bovine albumin solution with ^125^I-labeled human albumin into the airspaces (Rezaiguia et al., 1997). The concentration of unlabeled and labelled protein in the distal airspaces over 1h is used as an index of net AFC (Rezaiguia et al., 1997). Surprisingly, the investigators detect a *P. aeruginosa* pneumonia-induced increase in AFC over 1h by about 48% in experiments with blood flow, and by 43% in those without blood flow, compared with control groups (Rezaiguia et al., 1997). In both experiments, this AFC increase is inhibited by amiloride, which blunts ENaC activity. As propranolol has no inhibitory effect on AFC, a catecholamine-dependent mechanism can be excluded (Rezaiguia et al., 1997). Intratracheal instillation of an anti-TNF neutralizing antibody into the lungs 5 min before bacterial instillation is shown to prevent the increase in AFC in rats with pneumonia (Rezaiguia et al., 1997) and instillation of 5 mg TNF in normal rats increased AFC by 43% over 1h compared to control rats receiving vehicle (Rezaiguia et al., 1997), an effect not blunted by propranolol. As such, TNF at early time points following G^–^ pneumonia can upregulate net AFC, partly by a TNF-dependent mechanism (Rezaiguia et al., 1997). This study thus provides evidence of a positive role of TNF in AFC, in a catecholamine-independent manner.

Studies by Börjesson et al. (2000) in ventilated, anesthetized rats with 45 min intestinal ischemia followed by 3 h reperfusion show development of remote organ injury, including the lungs. A 76% increase in AFC as compared to controls, measured as the increase in alveolar protein concentration over 45 min, is at least partially mediated by a TNF-dependent mechanism, since systemic administration of a neutralizing polyclonal anti-TNF antibody before induction of intestinal ischemia fully inhibits the rise in AFC. Similar to the results above (Rezaiguia et al., 1997), the stimulated AFC after intestinal ischemia-reperfusion cannot be inhibited by propranolol and is therefore assumed to be catecholamine-independent (Börjesson et al., 2000). Intracellular cAMP levels are not increased in this model and amiloride inhibits similar fractions of AFC in animals subjected to ischemia-reperfusion or in control animals (Börjesson et al., 2000). In conclusion, the rat intestinal ischemia-reperfusion model leads to an AFC increase that is TNF-dependent.

Evaluation of AFC and epithelial and endothelial permeability in a model of chronic allergic inflammation in Brown-Norway rats challenged with ovalbumin by Tillie-Leblond et al., demonstrates lung sections with inflammatory infiltrates associated with perivascular and peribronchiolar edema in the ovalbumin group (Tillie-Leblond et al., 2002). To measure AFC in this study, a 5% bovine albumin solution with ^125^I-labeled human albumin is instilled into their air spaces and alveolar-capillary barrier permeability is evaluated in another group of animals by intravenous injection of 1 μCi of ^131^I-labeled albumin (Tillie-Leblond et al., 2002). Endothelial permeability in this study significantly increased in the ovalbumin group but the final-to-initial protein ratio reaches significantly higher levels in the ovalbumin group, as compared to the control group (Tillie-Leblond et al., 2002). Administration of neutralizing rat anti-TNF antibodies in the instillation significantly decreases this ratio. As such, also in this rat model of severe bronchial allergic inflammation associated with increased endothelial and epithelial permeability, a TNF-dependent increase in AFC is observed.

These three *in vivo* studies of G- pneumonia, intestinal ischemia and reperfusion or ovalbumin-induced inflammation give clear evidence of an increase in AFC involving a crucial role of TNF (Hamacher et al., 2018).

One of the explanations for the observed AFC-promoting actions of TNF discussed above can come from either positive actions of TNFR2 signaling or from a role for the lectin-like domain of TNF. Indeed, in sharp contrast to the TNFR1 binding site, the lectin-like domain of TNF, mimicked by the TIP peptide (a.k.a. Solnatide, AP301), can activate amiloride-sensitive Na^+^ uptake in alveolar epithelial cells, as well as in lung and glomerular MVEC, which also express all ENaC subunits (Hribar et al., 1999; Elia et al., 2003; Braun et al., 2005; Madaio et al., 2019). This effect of the lectin-like domain of TNF occurs even in cells lacking both TNF receptors (Hribar et al., 1999) thus clearly documenting that the TIP peptide binds to partners different from the TNF receptors. Actually, TIP peptide binds to the α subunit of ENaC and as such directly increases the channel’s activity. This occurs by affecting both the open probability (*Po*) and the cell surface (*N*) expression of ENaC (Czikora et al., 2014; Shabbir et al., 2015; Lucas et al., 2016). Treatment of cells with *N*-glycosidase F, which removes *N*-linked glycosylation from ENaC subunits, completely abrogates channel activation by the TIP peptide (Czikora et al., 2014). This interaction mainly affects the promotion of ENaC’s cell surface expression and involves the Asn511 residue in the extracellular loop of ENaC-α (Shabbir et al., 2015). The effects of the peptide on ENaC open probability rather involve a different interaction site within the second transmembrane domain of ENaC-α in which Val567 and Glu568 are crucial (Lucas et al., 2016). The TIP peptide even activates ENaC in the presence of bacterial toxins like pneumococcal pneumolysin, a pore-forming toxin which impairs both the *Po* (Lucas et al., 2012a) and *N* of ENaC (Lucas et al., 2016), both *in vitro* and *in vivo* (Czikora et al., 2014; Hamacher et al., 2018).

### Shifting the Balance Between the Tumor Necrosis Factor Receptor- and Lectin-Like Domain-Mediated Effects of Tumor Necrosis Factor

Despite of the negative effects of TNFR1 on ENaC, the lower affinity of TNF for oligosaccharides in glycoproteins than for its membrane-associated TNF receptors (Hession et al., 1987; Sherblom et al., 1988) and the much higher expression level of TNF receptors than of ENaC in alveolar epithelial cells, an intriguing observation is that inhibition of TNF with a neutralizing antibody worsened edema formation in a rat pneumonia model (Rezaiguia et al., 1997). An important question to ask is under what conditions can the beneficial activity of the lectin-like domain of TNF on ENaC predominate the deleterious one exerted by TNF-TNFR1 signaling? In an isolated perfused rat flooded lung model, which mimics cardiogenic (hydrostatic) edema, human TNF significantly decreases lung liquid clearance. Whereas the neutralizing anti-TNF monoclonal antibody Infliximab restores basal liquid clearance in this model, complex formation of TNF with its soluble TNF receptor 1 increases fluid clearance above basal levels, comparable to treatment of the lungs with TIP peptide (Braun et al., 2005). Whereas Infliximab likely interferes with both the receptor binding sites and the lectin-like domain of TNF, the soluble TNFR1 construct only blunts the former (Lucas et al., 1994; Braun et al., 2005). As such, it is tempting to hypothesize that the lectin-like domain of TNF can play a physiologically relevant role especially when present in complexes between TNF and its soluble TNF receptors, which blunt the receptor binding sites but leave the lectin-like domain active. This condition can occur in pneumonia, as such providing a potential explanation for the observed positive effects of TNF in a rat model of severe bacterial pneumonia (Rezaiguia et al., 1997). Triple mutant TNF mice expressing a mutant TNF lacking a functional lectin-like domain exhibit increased lung injury upon pneumolysin instillation as compared to wild type mice (Czikora et al., 2014). These findings indicate that the lectin-like domain can exert its protective role within the context of the entire TNF molecule and suggests that it represents an intramolecular negative feedback mechanism to keep a brake on TNF’s potent inflammatory activities.

### Barrier-Protective Activities of the Lectin-Like Domain of Tumor Necrosis Factor

**FIGURE 4:**
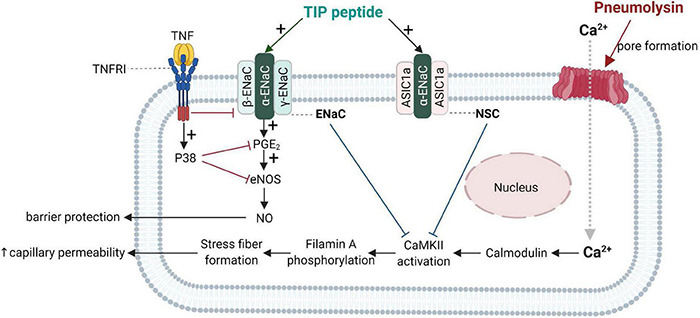
Barrier-protective mechanism mediated by the TNF lectin-like domain in pneumolysin-treated human lung microvascular endothelial cells [adapted from Czikora et al. (2017)]. Pneumolysin forms pores in cholesterol-containing cell membranes, which increases Ca^2+^ -influx (Lucas et al., 2012b), which mobilizes calmodulin to activate CaMKII, which in turn phosphorylates its substrate filamin A. Phosphorylated filamin A (Alli et al., 2015) then promotes stress fiber formation and enhances permeability of capillaries. Activation of ENaC by TIP peptide or of the non-selective cation channel, (NSC) either by TIP peptide (binding to ENaC-α) or MitTx (binding to ASIC1a), inhibits pneumolysin-mediated CaMKII activation and subsequent hyperpermeability. ENaC activation moreover promotes PGE2 generation and eNOS activation, resulting in increased barrier-protective NO production. ASIC1a, acid-sensing ion channel 1a; CaMKII, Calcium/calmodulin- dependent protein kinase II; ENaC, epithelial sodium channel.

Important progress in the understanding of the biological role of the lectin-like domain of TNF comes from *in vivo* pneumonia and ARDS models evaluating the potential therapeutic potential of the TIP peptide. As shown in Figure 4, both ENaC (in its native configuration α, β, γ) and the non-selective cation channel (NSC, consisting of an ASIC1a and an ENaC-α subunit), can be expressed in lung MVEC (Czikora et al., 2017). TIP peptide, which binds to ENaC-α, can strengthen barrier function in cells treated with the pore-forming bacterial toxins listeriolysin-O (LLO, from *Listeria monocytogenes*) and pneumolysin (PLY, from *Streptococcus pneumoniae*). Both LLO and PLY induce Ca^2+^-influx, which in turn activates Ca^2+^-dependent PKC isozymes, such as PKC-α, involved in capillary barrier dysfunction (Xiong et al., 2010; Lucas et al., 2012b). Both LLO and PLY impair ENaC activity (Lucas et al., 2012a; Yang G. et al., 2018). Our results show a profound protective effect of TIP peptide toward both LLO and PLY-induced barrier dysfunction, assessed by electrical cell substrate impedance sensing (ECIS), in human lung microvascular endothelial cell (MVEC) monolayers *in vitro* and in mice *in vivo*, the latter assessed by Evans Blue Dye incorporation (Xiong et al., 2010; Lucas et al., 2012b). A crucial role for ENaC-α in this process is documented by the significantly elevated sensitivity to PLY-induced barrier dysfunction in human lung MVEC in which ENaC-α is depleted by specific siRNA. Moreover, the protective effect of both the TIP peptide and of the NSC activator MitTx is lost under these conditions. TIP peptide blunts PLY-induced phosphorylation of calmodulin-dependent kinase II (CaMKII), as well as of its substrate filamin A (FLN-A) (Czikora et al., 2017). Non-phosphorylated FLN-A both promotes ENaC open probability and blunts stress fiber formation, thus providing a mechanism and a link for the ENaC activating and barrier protective role of the lectin-like domain of TNF.

Transplanted lungs often experience severe alveolar epithelial and endothelial dysfunction immediately following the procedure, due to ischemia-reperfusion injury. In the rat left-sided lung transplantation model of severe acute lung injury due to 20 h of cold ischemia and 24 h of reimplantation response, TIP peptide given intratracheally immediately before reimplantation into the left lung increases the left lung PaO_2_/FiO_2_ (P/F) ratio from about 70 mm Hg -indicating a condition similar to severe ARDS- to more than 400 mm Hg, which rather corresponds to a mild hypoxia-induced lung injury where a patient can typically walk up even stairs (Hamacher et al., 2010, 2018). Patient with a similar drop in P/F ratio as the one observed in the rat transplantation study group not treated with TIP peptide need to be tracheally intubated and are completely dependent on both the ventilator’s respiratory work and on a high fraction of oxygen supplementation. When applied together with the pharmacological ENaC inhibitor amiloride, the protective effect of the TIP peptide is abrogated. Complex formation of TIP peptide with *N, N*′-diacetylchitobiose, a selective oligosaccharide inhibitor of the lectin-like domain of TNF, or use of an inactive mutant TIP peptide, in which three crucial residues (1 Threonine and 2 glutamic acids) are changed for alanine also fail to restore oxygenation. These data strongly suggest that the significant protective effect of the TIP peptide in a severe ischemia-reperfusion lung injury model involves the lectin-like activity of TNF and is due to the activation of Na^+^ uptake via ENaC (Hamacher et al., 2010). The reduced infiltration of neutrophils in the bronchoalveolar lavage fluid, as well as the reduced generation of reactive oxygen species (ROS, discussed in the next paragraph), further underlines that the lectin-like domain of TNF can modify important inflammatory pathways in lung ischemia-reperfusion injury.

### Anti-oxidant and Anti-inflammatory Actions of the TIP Peptide

One of the main reasons for endothelial dysfunction following lung transplantation is the alteration in blood flow, which results in shear stress that can be sensed by a mechanosensitive complex -the mechanosome- in the endothelial cell membrane. The components of this mechanosome include caveolae, the adhesion molecule PECAM, vascular endothelial growth factor receptor 2 (VEGFR2) and the adherens junction protein VE-cadherin (Chatterjee and Fisher, 2014; Chatterjee et al., 2015). The shear signal can be transduced by components of the cell surface membrane, resulting in the activation of NADPH oxidases and the generation of ROS. In a rat iso-transplantation model, a significantly increased ROS generation occurs in the transplanted lung 24h after the procedure, but this is prevented in by pretreatment of the transplanted lungs with TIP peptide, but not with an inactive mutant peptide. This ROS-reducing activity of the TIP peptide correlates with by a significantly reduced infiltration of neutrophils in the bronchoalveolar lavage fluid (Hamacher et al., 2010). Both of these effects can be blunted by the ENaC inhibitor amiloride. TIP peptide also blunts ROS generation *in vitro* in ovine pulmonary artery endothelial cells undergoing hypoxia-reoxygenation. The results from these preclinical studies suggest that the TIP peptide is a therapeutic candidate to treat ischemia-reperfusion injury associated with lung transplantation, since it can blunt both oxidative stress and inflammation in endothelium.

Another study documenting the anti-inflammatory capacity of the TIP peptide comes from a study using a rat model of high-altitude pulmonary edema (HAPE). In this study, treatment with TIP peptide (a.k.a. Solnatide, AP301) reduces leukocyte infiltration and pro-inflammatory cytokine levels in lavage, while improving occludin expression in alveolar epithelial cells and reducing edema formation (Zhou et al., 2017).

**FIGURE 5:**
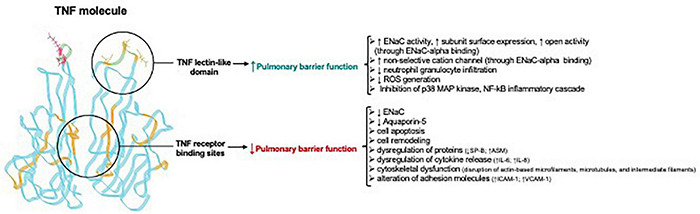
Divergent actions of TNF receptor binding sites versus the lectin-like domain in acute lung injury Adapted from Hamacher et al. (2018). Note that the TNF receptor binding sites for TNF receptor type 1 and TNF receptor type 2 are in the regions between two TNF homotrimers and are not identical, but in part overlapping. ↑, upregulation; ↓, downregulation; ASM, acid sphingomyelinase; ENaC, Epithelial sodium channel; ICAM-1, intercellular adhesion molecule 1; Na^+^/K^+^ -ATPase, sodium–potassium adenosine triphosphatase; VCAM-1, vascular cell adhesion molecule-1; ROS, reactive oxygen species.

The TIP peptide also has an anti-inflammatory action in other organs than the lungs, as shown in a mouse glomerulonephritis model, in which animals were treated with nephrotoxic serum. In this model, local immune deposits in the glomeruli generate TNF, which activates pro-inflammatory pathways in both glomerular endothelial cells and podocytes. Intraperitoneal treatment with TIP peptide in this model significantly reduces inflammation (reduced systemic and local pro-inflammatory cytokine generation, and reduced renal leukocyte infiltration), as well as proteinuria and blood urea nitrogen (Madaio et al., 2019). TIP peptide also increases PGE_2_ generation in glomerular endothelial cells, which in turn promotes eNOS-dependent NO generation and barrier function (Figure 4). Despite the capacity of TIP peptide to activate ENaC, it does not increase mean arterial blood pressure in mice, possibly because it is captured in the loops of Henle by Tamm-Horsfall protein (Hession et al., 1987; Sherblom et al., 1988) before it can reach the distal tubules of the kidney. Figure 5 and Table 2 give an overview of the divergent actions of the TNF receptor binding sites *versus* the lectin-like domain in models of acute lung injury.

**TABLE 2:** Divergent actions of TNF via its receptor binding sites *versus* its lectin-like domain in the lungs

Target	TNF receptor binding sites	Lectin-like domain of TNF
**Cell viability**	- Apoptosis in epithelial cells in mouse acute lung injury model [involving TNF-TNFR1- > caspase-8 and caspase 3 activation pathway (Patel et al., 2013)] - Induction of apoptosis in primary endothelial cells (Robaye et al., 1991)	Not investigated
**Adhesion molecules**	- TNF induced ICAM-1 expression in human vascular endothelial and lung epithelial cells (Burke-Gaffney and Hellewell, 1996) and in lung airway epithelium (Kim et al., 2008) - TNF causes redistribution of p120 catenin and E-cadherin from adherens junctions in primary bronchial epithelial cells (Hardyman et al., 2013) - ↑ ICAM-1 and ↑ VCAM-1 expression inhuman pulmonary endothelial cells (Zhou et al., 2007; Su et al., 2008; Proudfoot et al., 2018)	Not investigated
**Non-selective cation channel**	Not investigated	Activation of non-selective cation channel in pneumolysin-treated HL-MVEC monolayers (Czikora et al., 2017)
**Water channel**	↓ aquaporin-5 expression in mouse lung (TNF-TNFR1- > NF-κB activation) (Towne et al., 2000; Towne et al., 2001)	Not investigated
**Leucine-rich repeat-containing 8A channel**	Inhibition of the activity of leucine-rich repeat-containing 8A in ATII cells (Zhang et al., 2019)	Not investigated
**ENaC/Na^+^ uptake**	↓ ENaC mRNA expression and activity in rat AT II cells (TNF-TNFR1- > NF-κB activation) (Dagenais et al., 2004; Yamagata et al., 2009)	- ↑ ENaC activity (Rezaiguia et al., 1997; Fukuda et al., 2001; Tillie-Leblond et al., 2002; Hazemi et al., 2010; Shabbir et al., 2013; Czikora et al., 2014; Shabbir et al., 2015; Willam et al., 2017a,b) - ↑ ENaC surface expression (Czikora et al., 2014; Shabbir et al., 2015) - ↑ open probability of ENaC (Hazemi et al., 2010; Shabbir et al., 2013; Czikora et al., 2014; Shabbir et al., 2015; Lucas et al., 2016) - ↑ Na^+^ current in primary dog, pig and rat ATII cells (Tzotzos et al., 2011) - ↑ amiloride-sensitive Na^+^ uptake in ATIIs (Vadasz et al., 2008; Hamacher et al., 2010) - ↑ increase in amiloride-sensitive Na^+^ uptake in mouse lung MVEC lacking both TNF receptors. TNFR1 and TNFR2 indicating the lectin-like activity of TNF increases AFC (Hribar et al., 1999)
**Release of inflammatory markers**	- ↑ IL-6 in mouse alveolar epithelial cells (TNF-TNFR1- > NF-κB activation) (Schwingshackl et al., 2013) - ↑ IL-8 *in vitro* model using A549 cell line (Standiford et al., 1990; Kwon et al., 1994) - ↑ IL-8 in human lung microvascular endothelial cells (TNF-TNFR1/p38 MAPK/MK2) (Su et al., 2008) - Activation of NADPH oxidases, resulting in ROS generation and consecutive degradation of IκBα in primary human pulmonary artery endothelial cells (TNF-TNFR/PI3Kγ/PKC-ζ/NOX) (Frey et al., 2006)	- ↓ levels of TNF, IL-1b, IL-6, and IL-8 in the blood of animals treated with medium doses of TNF-lectin-like domain (Zhou et al., 2017) - ↓ IL-6, TNF, cyclooxygenase-2, tenascin-c in porcine model of systemic sepsis-associated lung injury (Hartmann et al., 2015) - ↓ PMN infiltration after lung transplantation (Hamacher et al., 2010) - ↓ hypoxia-induced ROS generation in ovine pulmonary artery endothelial *cells in vitro* and blunts ischemia- reperfusion-induced ROS production in transplanted rat lungs *in vivo* (Hamacher et al., 2010)
**Lung function & pulmonary edema/Acute lung injury**	- ↑ pulmonary edema (Yang et al., 2010) - Inhibition of edema reabsorption *in vivo* flooded rat lung model and *in situ* rat model (Braun et al., 2005) - ↑ pulmonary edema *in situ* mouse model and *ex vivo* rat model (Elia et al., 2003) - Initiation of acute inflammation and edema formation (Fukuda et al., 2001)	- ↓ pulmonary edema,, ↓ BAL fluid protein in high-altitude control rats and improved gas-blood barrier function during acute hypobaric hypoxia and exercise in rats (Zhou et al., 2017) - ↓ extravascular lung water index in patients with SOFA scores ≥ 1indicated by an exploratory post-hoc subgroup analysis in a phase IIa randomized placebo-controlled trial (Krenn et al., 2017)
	- Induction of acute lung leak in rats (Koh et al., 1996) - TNF release in endotoxemia contributes to neutrophil-dependent pulmonary edema (Horgan et al., 1993) - TNF mediates experimental pulmonary edema by ICAM-1 and CD18-dependent mechanisms (Lo et al., 1992) - Induction of pulmonary edema *in vivo* guinea pig model (Hocking et al., 1991) - Induction of pulmonary edema in the isolated guinea pig lung (Hocking et al., 1990)	- Normalization of both sodium and fluid absorption in oedematous alveoli to non-oedematous levels (Shabbir et al., 2015) - Oral inhalation of AP301 Activation of pulmonary edema clearance in a phase IIa clinical trial in mechanically ventilated ICU patients (Krenn et al., 2014) - No effect on pneumovirus-induced pulmonary edema in mice (van den Berg et al., 2014) it as a negative study in a model not known to have positive controls - TNF tip peptid/TIP improved lung oxygenationin/functionan *in vivo* setting of IR injury associated with isotransplantation in female Wistar rats from severest gas exchange disturbance equivalent to severe ARDS to near-normal lung function (Hamacher et al., 2010) - Activation of ALC indicated in several animal models of hydrostatic and permeability edema (Braun et al., 2005; Vadasz et al., 2008; Lucas et al., 2012a; Hartmann et al., 2013) - Activation of ALC in *ex vivo* model of blood-perfused isolated flooded rat lung and in TNFR1/R2/C57BL/6 mice (Elia et al., 2003) - ↑ AFC in ventilated rats (Fukuda et al., 2001) - ↑ Resolution of alveolar edema in rats and mice (Elia et al., 2003) - ↑ Stimulation ALC in intestinal ischemia-reperfusion in rats (Börjesson et al., 2000)
**Barrier integrity**	- Induction of lung endothelial cell barrier disruption in endotoxin-induced pulmonary edema (Yu et al., 2016; Zhang et al., 2019) - Induction of cytoskeletal changes, paracellular gap formation, and increased permeability to fluxes of dextran and albumin in human pulmonary microvascular endothelial cells (Koss et al., 2006) - Increased endothelial permeability in pulmonary artery endothelial cells, by means of destabilizing microtubules, which in turn induces barrier dysfunction in a RhoA/ROCK-dependent manner (Petrache et al., 2003) - ↑ epithelial permeability that occurs during *Corynebacterium parvum*-induced acute alveolitis (Li et al., 1995) - ↓ ZO-1, Claudin-2, Claudin-4, Claudin-5 and β-catenin carrageenan-induced acute lung inflammation of mice (Mazzon and Cuzzocrea, 2007) - ↓ Occludin and claudins *in vitro* in differentiated human bronchial epithelial cultures (Hardyman et al., 2013) - Microtubule destabilization, actin rearrangement, and endothelial barrier dysfunction in human pulmonary artery endothelial cells by p38 mitogen-activated protein kinase activation (Petrache et al., 2003)	- Improving gas exchange (Aigner et al., 2017) - ↑ occludin expression in high-altitude control rats and improving gas-blood barrier function during acute hypobaric hypoxia and exercise in rats (Zhou et al., 2017) - Strengthened barrier function in PLY treated HL-MVEC by binding to ENaC-α, and the specific ASIC1a activator MitTx (Czikora et al., 2017) - Restoration of impaired endothelial barrier function in the presence of the pore-forming toxins PLY and listeriolysin-O (Xiong et al., 2010; Lucas et al., 2012a) - Blunts listeriolysin-O and pneumolysin-induced hyperpermeability in HL-MVEC (Xiong et al., 2010) - ↑ barrier integrity/function in a rat model of high altitude pulmonary edema (Zhou et al., 2017) - Improvement of alveolar fluid balance by both reducing vascular permeability and enhancing the absorption of excess alveolar fluid in experimental in rabbit lung injury model (Vadasz et al., 2008)
**Lactate dehydrogenase**	- Dose-dependent increase in lactate dehydrogenase release and number of detached cells for cells of the pulmonary artery (Meyrick et al., 1991)	Not investigated

*↑, upregulation/increase; ↓, downregulation/decrease; AECs, alveolar epithelial cells; ALC, Alveolar liquid clearance; ALI, Acute lung injury; ATII, Alveolar type II cells; ENaC, epithelial sodium channel; HL-MVEC, human lung microvascular endothelial cells; ICAM-1, intercellular adhesion molecule 1; IL, Interleukin; NF-κB, Nuclear factor-κB; NOX, NADPH oxidase; PKC, Protein kinase C; ROS, reactive oxygen species; TNF, tumor necrosis factor; TNFR, tumor necrosis factor receptor; TIP, TNF Tip peptide (17 amino acid circular peptide of TNF, stimulating TNF lectin-like region); VCAM-1, vascular cell adhesion molecule 1; ZO-1, Zona occludens-1.*

## Clinical Trials With the Tip Peptide in Patients With Acute Respiratory Distress Syndrome and Following Lung Transplantation

In view of promising data resulting from preclinical studies of TIP peptide performed by others and our group in several animal species, including mice, rats, rabbits and pigs (Elia et al., 2003; Braun et al., 2005; Hamacher et al., 2010; Madaio et al., 2019), clinical trials assessing safety and efficacy of TIP peptide (a.k.a. Solnatide, AP301) organized by APEPTICO, Vienna, Austria have been conducted at the Hospital of the Medical University Vienna, Austria. A double-blind placebo-controlled phase I study evaluating spirometry, exhaled NO, vital signs, ECG, safety laboratory and quantification of peptide in plasma, associated with six ascending inhaled doses of the TIP peptide in 48 healthy male subjects (Schwameis et al., 2014) demonstrates no serious, local, or dose-limiting adverse events with very low systemic exposure levels to the peptide. Subsequently, two double-blind, placebo-controlled phase 2a clinical trials in 40 patients with acute lung injury (ALI) (Krenn et al., 2017) and in 20 lung transplantation patients (Hamacher et al., 2010; Aigner et al., 2017; Ware, 2017) further document safety and efficacy. The main parameters in these studies are extravascular lung water index (EVLWI) -indicative of pulmonary edema and measured with the Picco method- (ALI trial) and days on the ventilator in the latter. In both the acute lung injury trial and the lung transplantation trial, patients receive 2 doses of the peptide *versus* placebo in the mechanical ventilator over seven days. Although no significant reduction in EVLWI occurs with TIP peptide inhalation over the entire patient population, it is interesting to note that there is a positive effect in a sub-group of patients with a sequential organ failure assessment (SOFA) score >11 (representing more than half of the study population), accompanied by a reduced ventilation pressure (Krenn et al., 2017). In the lung transplant patient group, a significant reduction in days on the ventilator is observed in patients receiving the peptide (Aigner et al., 2017). In 2016, TIP peptide obtains orphan drug designation for acute lung injury (from EMA), for lung transplantation (FDA and EMA) and for high-altitude pulmonary edema (HAPE) (FDA and EMA) (Hamacher et al., 2018). A phase 2b dose escalation study with TIP peptide in ARDS patients (a.k.a. Solnatide, AP301) is currently being conducted in eight university hospitals in Germany and Austria (ClinicalTrials.gov Identifier: NCT03567577](Schmid et al., 2021). Moreover, a phase 2a clinical trial in moderate to severe COVID-19 patients is being conducted at the Medical University Vienna (EudraCT 2020-001244-26).

Results obtained in the clinical trials so far document the translational and therapeutic potential of the TIP peptide -mimicking the lectin-like domain of TNF- for the treatment of alveolar-capillary dysfunction in ARDS and ischemia reperfusion injury after transplantation.

## Conclusion

Tumor necrosis factor is a highly complex dual mediator, harboring within its structure spatially distinct domains that can act either in a deleterious or a protective manner in ARDS (Braun et al., 2005; Czikora et al., 2014; Hamacher et al., 2018).

At high concentrations, the role of TNF in the regulation of lung function during pneumonia, ARDS and lung transplantation is traditionally thought to be mainly deleterious, mediated by TNF-TNFR1 signaling and characterized by increased alveolar epithelial and capillary endothelial cell demise, impaired AFC, barrier dysfunction, oxidative stress and hyper-inflammation. The situation is, however, far more complex. First, in certain pathological conditions, such as in ventilator-induced lung injury, the two TNF receptor types can mediate divergent effects upon binding TNF, with TNFR1 being deleterious and TNFR2 rather protective. Second, the lectin-like domain of TNF which is spatially distinct from the receptor binding sites exerts largely protective activities in ARDS and severe pneumonia upon binding to ENaC-α. These protective activities include activation of alveolar fluid clearance, strengthening of alveolar-capillary barrier function, and reduction of inflammation including a lesser production of tissular reactive oxygen species. The lectin-like domain of TNF is with high probability physiologically relevant, since it is clearly active in complexes between TNF and its soluble TNFRs (Braun et al., 2005). Moreover, mice expressing a mutant TNF lacking a functional lectin-like activity have increased lung injury upon bacterial toxin instillation (Czikora et al., 2014).

How the TNF-TNF receptor 1 and TNF receptor 2 actions on the one hand and the TNF lectin-like domain actions on the other hand interact with one another within the TNF homotrimer remains largely unknown and represents an important field of research in the forthcoming years. Not much is known with regard to the contribution of the receptor binding sites *versus* the lectin-like domain during the evolution of lung injury, its repair or remodeling and concerning mediator co-signaling, alveolar oncotic pressure increase, tissue hypoxia, and many others (Hamacher et al., 2018). Our data suggest that TNF contains within its tertiary structure a negative feedback mechanism able to put a brake on and to even counteract TNF-TNFR-mediated inflammation, as well as barrier and AFC dysfunction in the lungs. The concept to compare outcomes on lung function of clinical trials with neutralizing anti-TNF antibodies *versus* soluble TNF receptor constructs seems us to be of high interest, since only the latter approach would not interfere with the activities of the protective lectin-like domain of TNF.

The TNF lectin-like region, mimicked by the TIP peptide (a.k.a. Solnatide, AP-301), is showing promising outcomes in recent clinical trials in ARDS and lung transplantation and may represent a prototype for alternative therapeutic strategies, apart from TNF inhibitory antibodies or soluble receptor constructs, to blunt the harmful, but retain the potentially beneficial activities of TNF required in host defense in acute lung injury and ARDS, as well as in bacterial and viral pneumonia, including COVID-19 pneumonia. It should probably further be studied in important clinical settings where thus far no specific treatment exists, such as in idiopathic lung fibrosis.

Much experimental and clinical work has to be done for scientists and clinicians to better understand the role of TNF in lung diseases such as ARDS, in order to be able to therapeutically modulate the cytokine and its associated inflammatory disorders and in order to improve patient outcome and as such health care. Novel mediators and pathways -like those related to the lectin-like domain of TNF- might contribute to this endeavor.

## Author Contributions

JH, YH, DE, MR, and RL wrote the manuscript. RL, GC, RC, HH, SS, AL, MH, DA, MU-F, TF, TC, AV, DE, MR, and JH contributed to studies reported in the review. PE, DA, MU-F, TF and TC assisted with redaction of the manuscript for clarity and with the figures. All authors contributed to the article and approved the submitted version.

## Conflict of Interest

RL is inventor of several patents relating to the use of the TNF-derived TIP peptide in pulmonary edema reabsorption. The remaining authors declare that the research was conducted in the absence of any commercial or financial relationships that could be construed as a potential conflict of interest.

## Publisher’s Note

All claims expressed in this article are solely those of the authors and do not necessarily represent those of their affiliated organizations, or those of the publisher, the editors and the reviewers. Any product that may be evaluated in this article, or claim that may be made by its manufacturer, is not guaranteed or endorsed by the publisher.
